# Author Correction: National genomic surveillance integrating standardized quantitative susceptibility testing clarifies antimicrobial resistance in Enterobacterales

**DOI:** 10.1038/s41467-024-45349-1

**Published:** 2024-01-26

**Authors:** Shizuo Kayama, Koji Yahara, Yo Sugawara, Sayoko Kawakami, Kohei Kondo, Hui Zuo, Shoko Kutsuno, Norikazu Kitamura, Aki Hirabayashi, Toshiki Kajihara, Hitomi Kurosu, Liansheng Yu, Masato Suzuki, Junzo Hisatsune, Motoyuki Sugai

**Affiliations:** Antimicrobial Resistance Research Center, National Institute of Infectious Diseases, Tokyo, Japan

**Keywords:** Antimicrobial resistance, Bacterial genomics

Correction to: *Nature Communications* 10.1038/s41467-023-43516-4, published online 5 December 2023

The original version of the Supplementary Information file contained an error in Supplementary Figure 12b; the number of E. coli isolates in the figure was listed as 1422, where it should be 1462. This has been corrected in the PDF version of the Supplementary Information file.

The original version of the article contained an error on page 5 of the PDF; the published text reads “Among the 2314 ESBL strains (Fig. 1), 1484 were *E. coli* and 723 were *K. pneumoniae* strains at the MLST level” whereas this should instead read “Among the 2314 ESBL strains (Fig. 1), 1488 were *E. coli* and 727 were *K. pneumoniae* strains at the MLST level”. This has been corrected in the HTML and PDF versions of the article.

### Supplementary information

Supplementary Information

