# Supplementary material for: National genomic surveillance integrating standardized quantitative susceptibility testing clarifies antimicrobial resistance in Enterobacterales
Source: Nat Commun. 2023 Dec 5;14:8046. doi: 10.1038/s41467-023-43516-4 (PMC10698200; doi:10.1038/s41467-023-43516-4)
Supplement: Supplementary file 1 — Supplementary Information [file 41467_2023_43516_MOESM1_ESM.pdf]

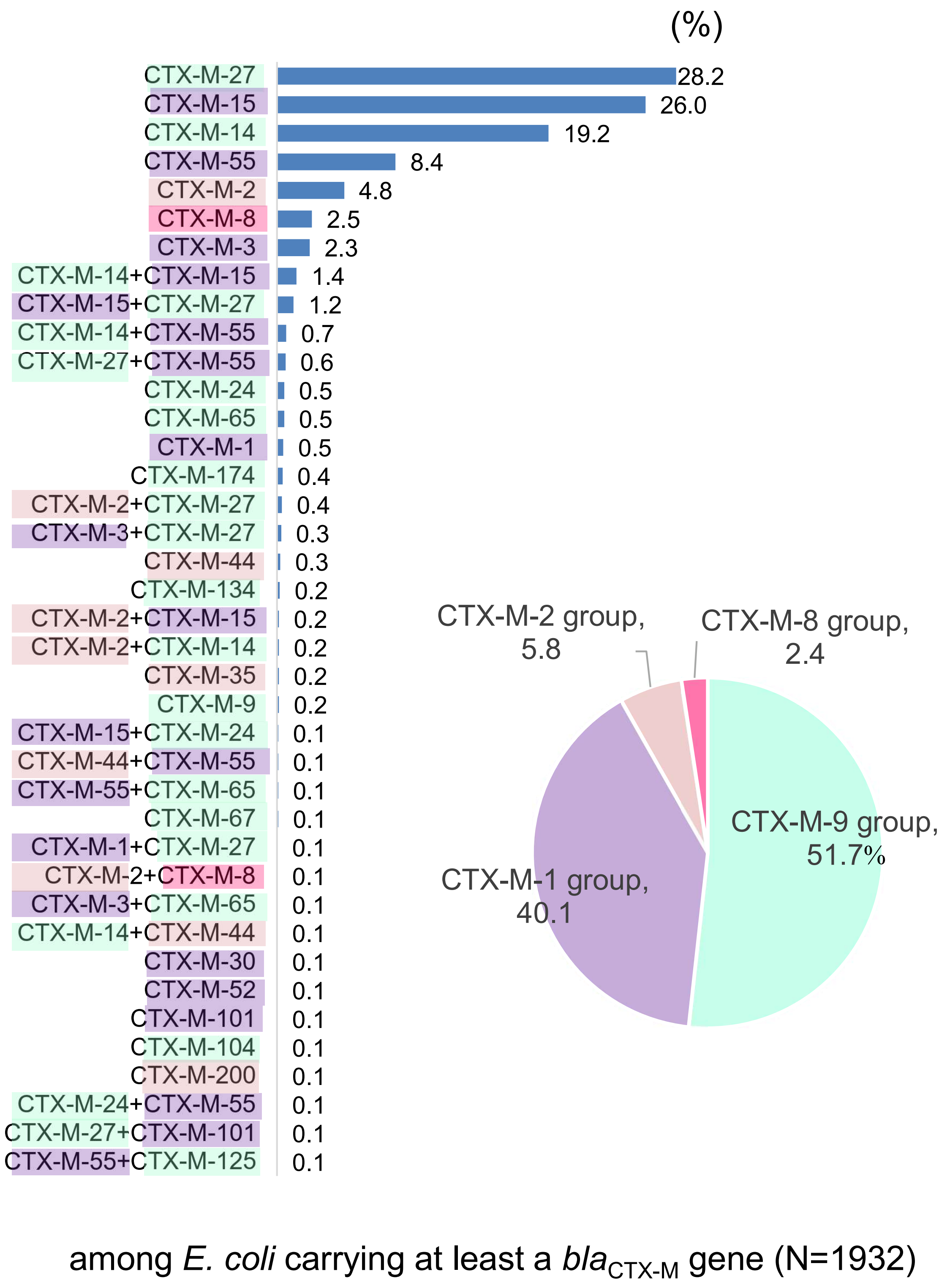

Supplementary Figure 1. Breakdown of bla_{CTX-M} genes among *E. coli* carrying at least a bla_{CTX-M} gene. The four colors on the left side of the bar plots represent four different bla_{CTX-M} groups.

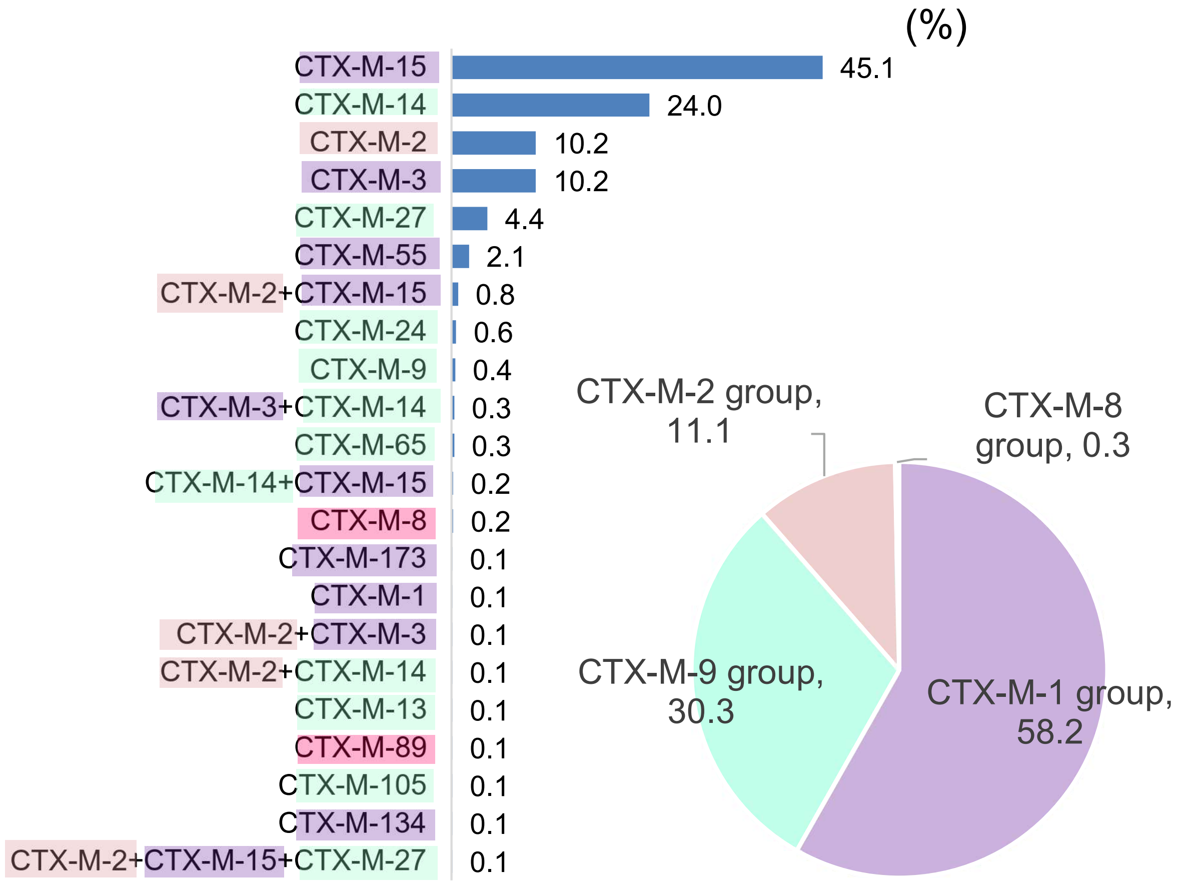

among *K. pneumoniae* carrying at least a *bla*$_{\text{CTX-M}}$ gene (N=890)

Supplementary Figure 2. Breakdown of *bla*$_{\text{CTX-M}}$ genes among *K. pneumoniae* carrying at least a *bla*$_{\text{CTX-M}}$ gene. The four colors on the left side of the bar plots represent four different *bla*$_{\text{CTX-M}}$ groups.

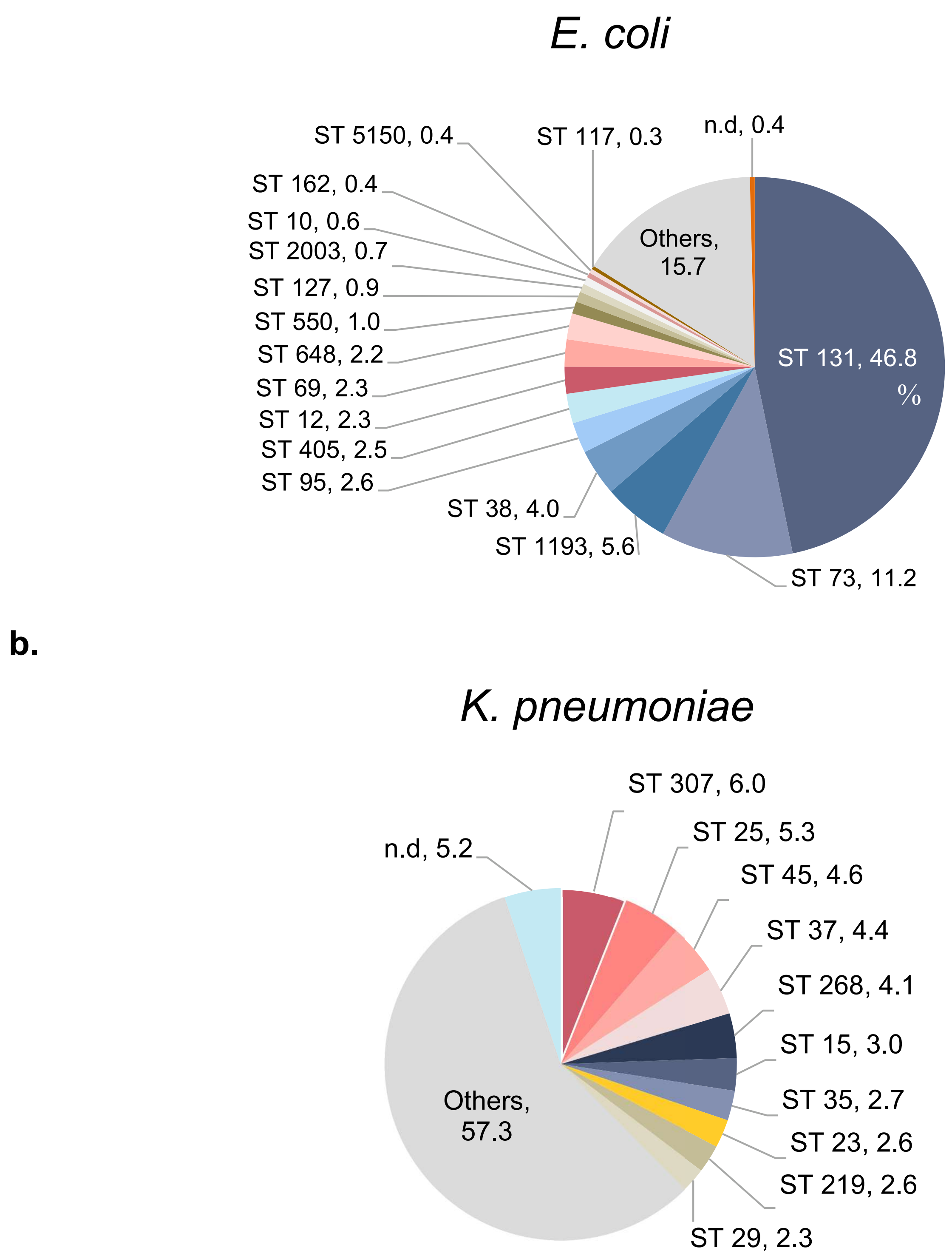

Supplementary Figure 3. Breakdown of STs among (a) *E. coli* (N=3159) and (b) *K. pneumoniae* (N=1240). n.d. stands for 'not determined.

E. coli

Supplementary Figure 4

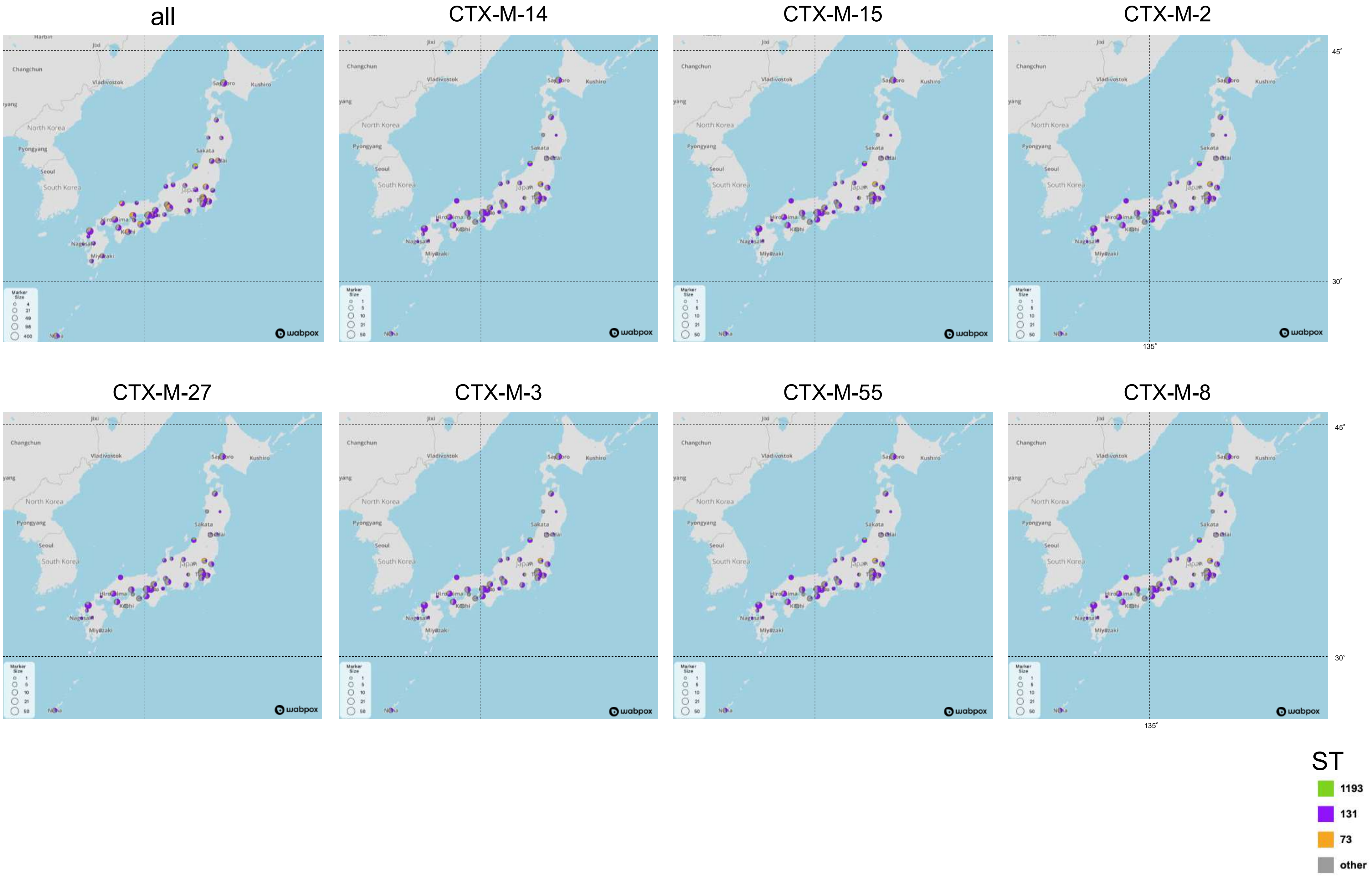

Supplementary Figure 4. Geographical distribution of *E. coli* based on sequence types (STs) and possession of *bla*$_{CTX\text{-}M}$ in Japan. The dashed lines represent latitudes and longitudes. Distribution of 3,158 *E. coli* isolates implemented WGS in this study. The maps were generated using Microreact, which utilizes Mapbox (text attribution: © Mapbox, © OpenStreetMap, and Improve this map). Interactive view and epidemiological and genotypic dataset (Inpatient/outpatient, clinical department, origin of sample, QRDR (amino acid position of *gyrA* 51, 67, 81, 82, 83, 84, 87, 106, *gyrB* 426, 447, *parC* 78, 80, 84, *parE* 445), results of antibiotic susceptibility test (S; susceptible, I; intermediate, R; resistant of ABPC/SBT, PIPC/TAZ, CMZ, CPDX, CTX, CTRX, CAZ, AZT, CFPM, IPM, MEPM, DRPM, GM, TOB, AMK, LVFX, CPFX, MINO, ST, CL, CP), MLST, possession of beta-lactamase genes (*bla*$_{IMP}$, *bla*$_{KPC}$, *bla*$_{NDM}$, *bla*$_{OXA}$ (carbapenemase or others), *bla*$_{VIM}$, *bla*$_{CTX\text{-}M}$, *bla*$_{SHV}$, *bla*$_{TEM}$, *ampC*) and *mcr* genes) are available at https://microreact.org/project/piQLyJmufXmM7gwCYzw6eN-microreactecoli3158up2022-04-25

K. pneumoniae

Supplementary Figure 5

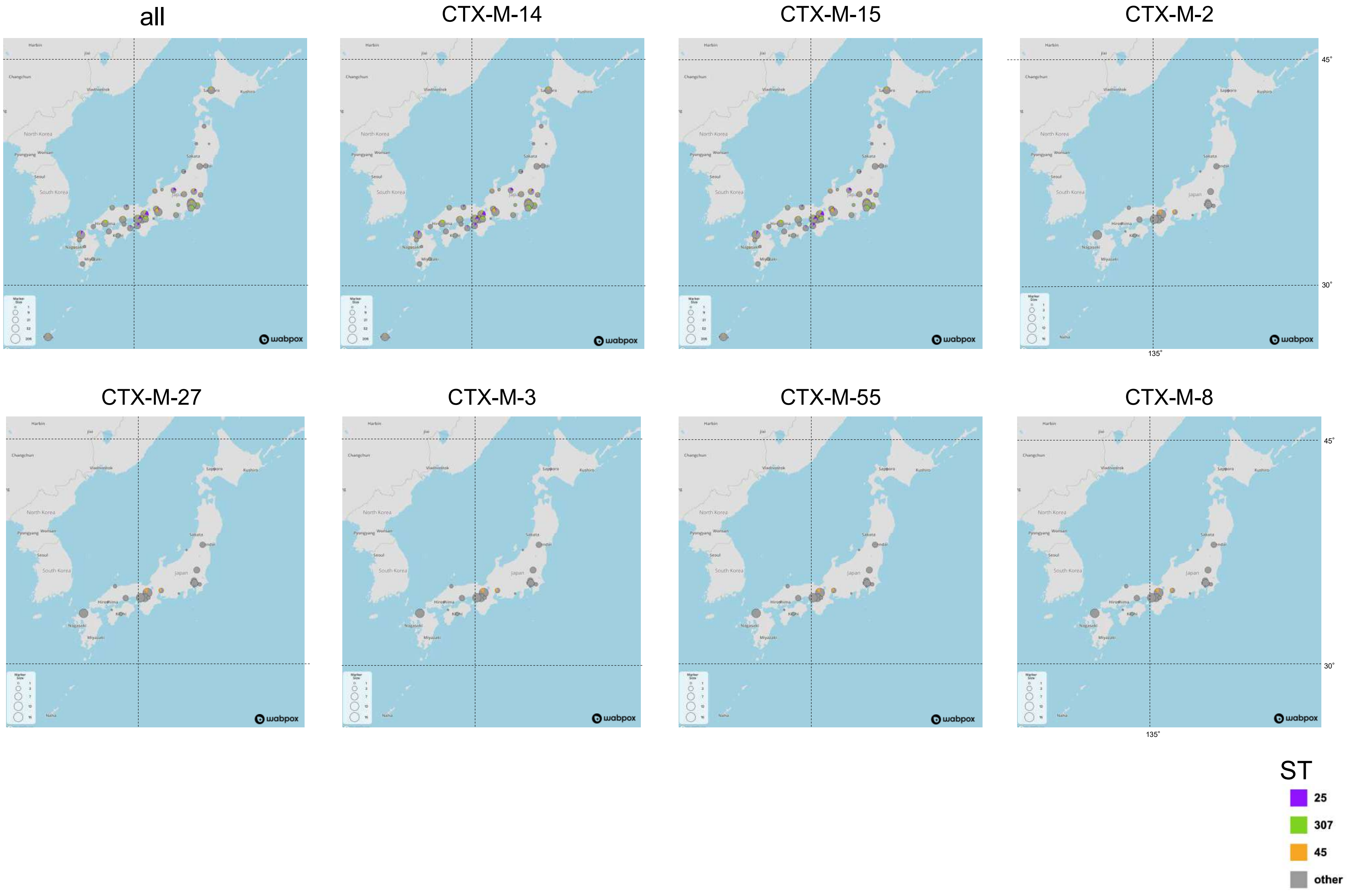

Supplementary Figure 5. Geographical distribution of *K. pneumoniae* based on sequence types (STs) and possession of *bla*$_{CTX-M}$ in Japan. The dashed lines represent latitudes and longitudes. The maps were generated using Microreact, which utilizes Mapbox (text attribution: © Mapbox, © OpenStreetMap, and Improve this map). Epidemiological and genotypic dataset of 1,240 *K. pneumoniae* isolates implemented WGS in this study. Interactive view and the full data of these isolates are available at https://microreact.org/project/2RRmtHGa74444NZHPivC4h-microreactkp1240up2022-04-26

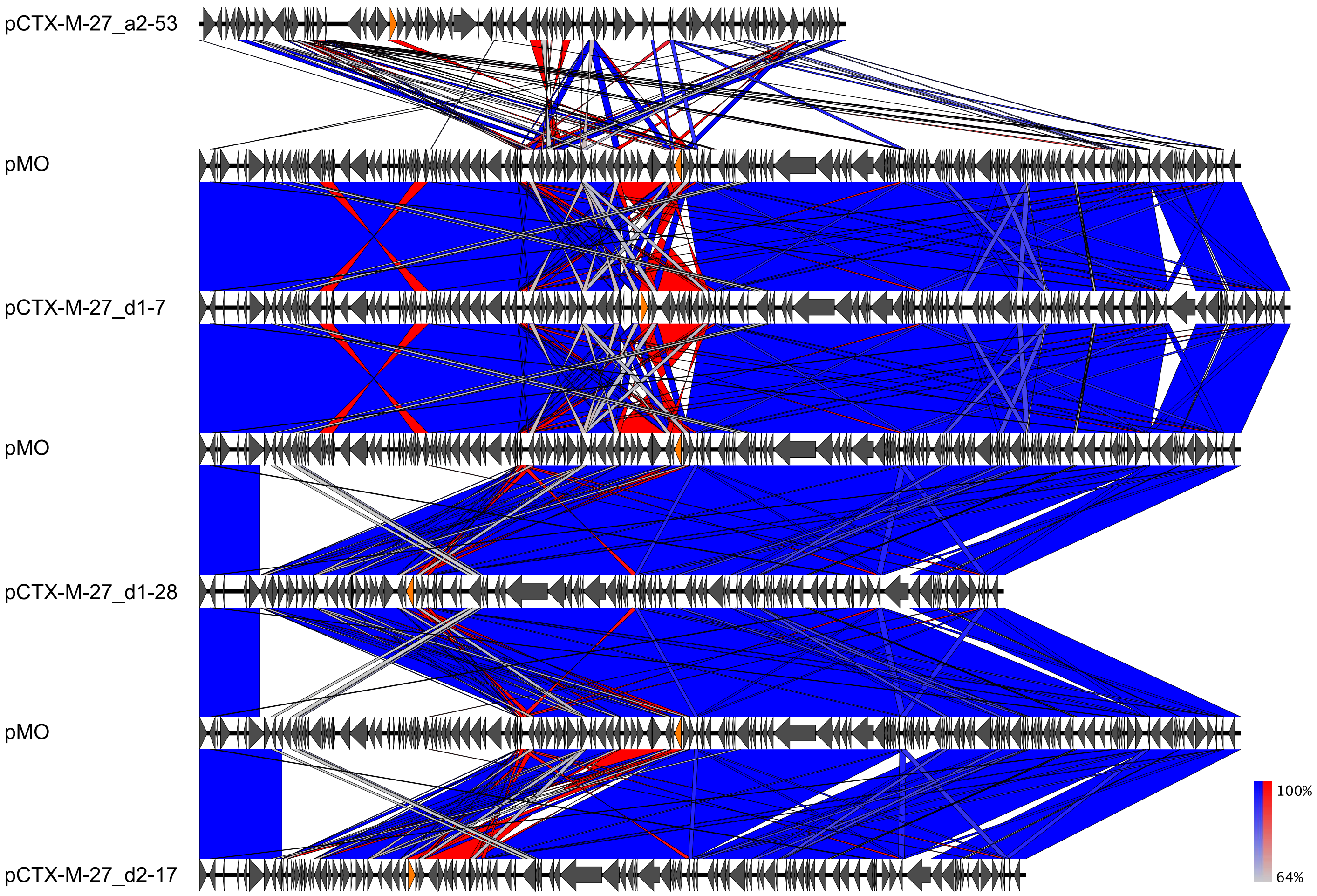

Supplementary Figure 6. Pairwise alignment between a known large transferable IncF1:A2:B20 plasmid associated with *bla*$_{\text{CTX-M-27}}$ (pMO) and each of reference plasmids used in this study.

Each reference plasmid and pMO are shown alternately. The *bla*$_{\text{CTX-M-27}}$ genes are highlighted with orange arrows. Blue signifies a high level of homology, while red signifies homology among complementary strands. Except for one of the reference plasmids at the top, overall structural similarity was observed.

CTX-M-15

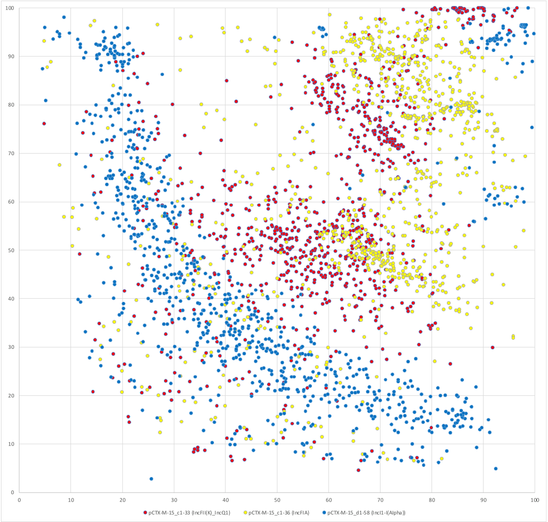

CTX-M-2

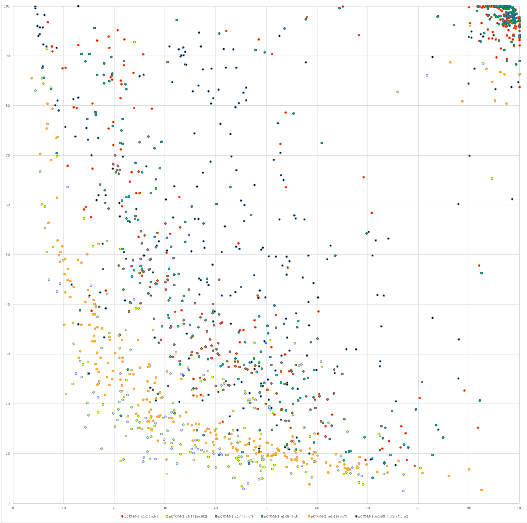

CTX-M-14

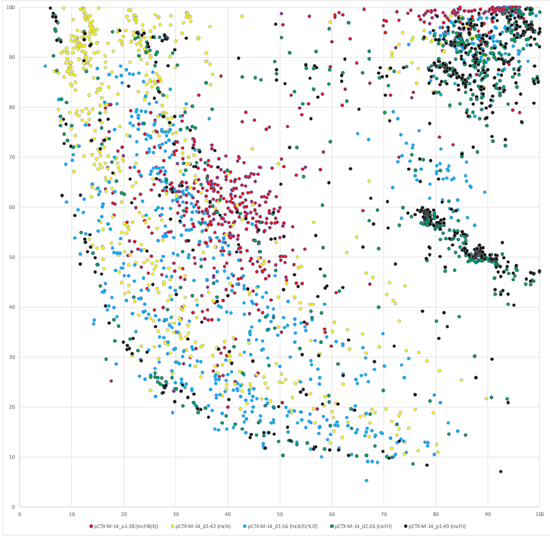

CTX-M-3

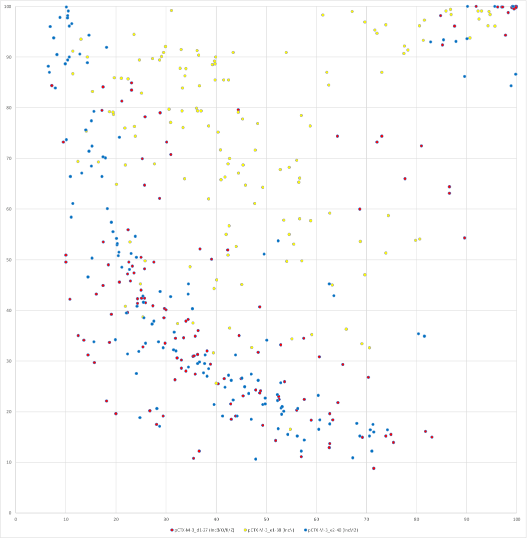

CTX-M-55

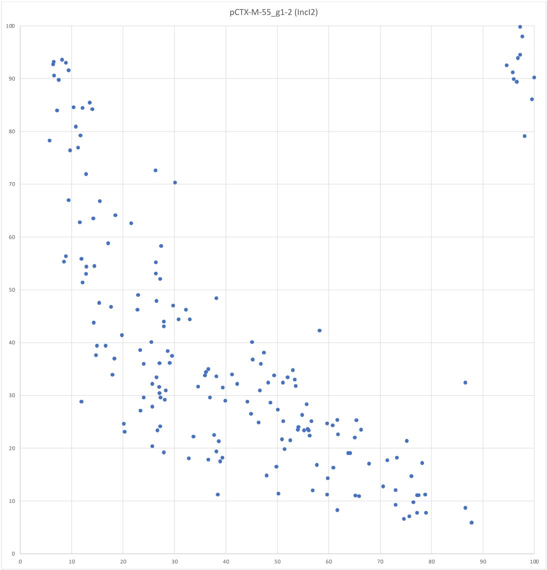

Supplementary Figure 7. The extent to which reference complete plasmid sequences can be aligned to draft genomes including a single *bla*$_{\text{CTX-M}}$ gene.

The x-axis indicates the percentage of total aligned sequence length; the y-axis indicates sequence identity in the aligned sequences. Colors represent different reference plasmids plotted for each isolate. Source data are provided as a Source Data file.

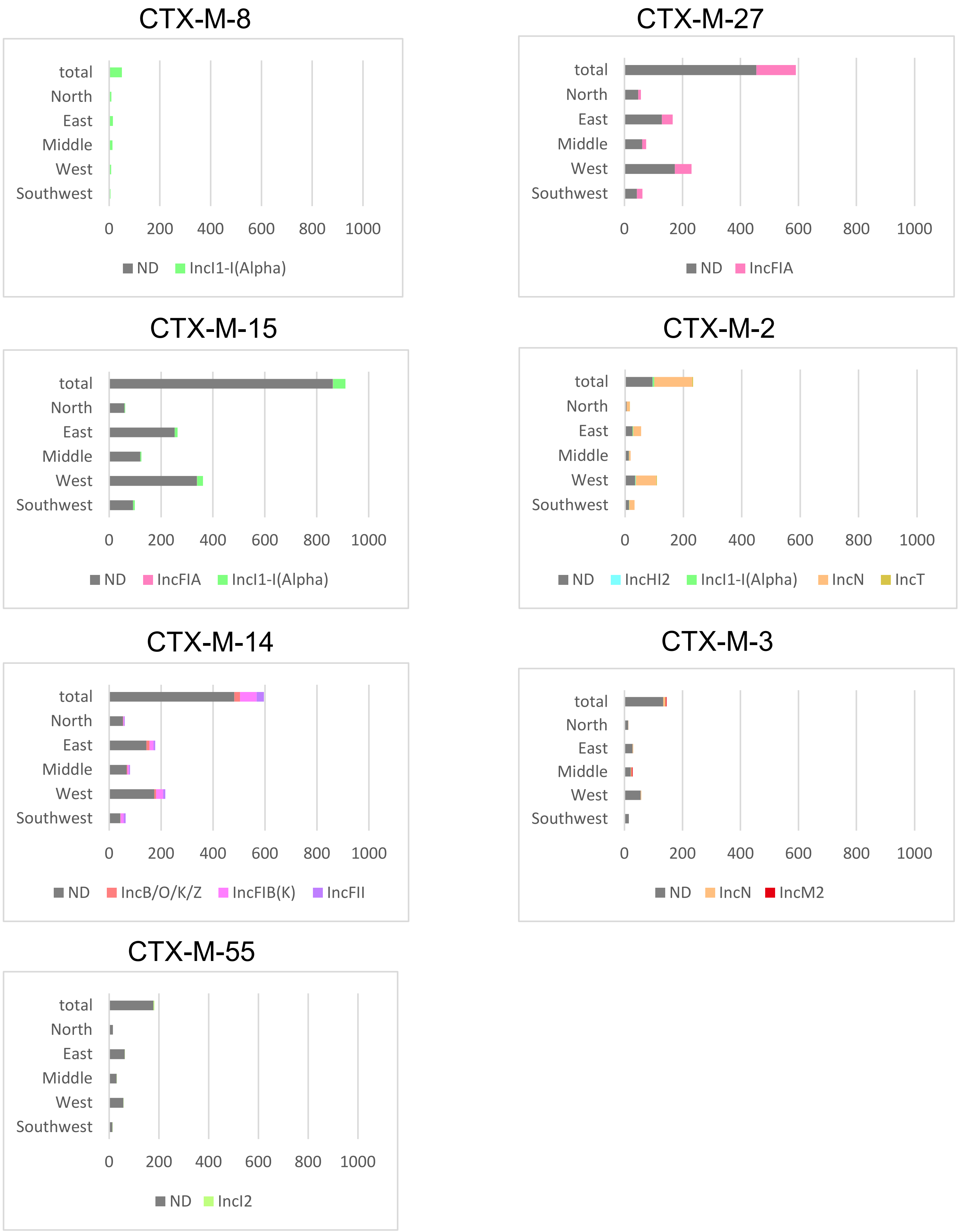

Supplementary Figure 8. Geographical differences in the proportion of strains well aligned by the reference complete plasmid sequences encoding *bla*$_{CTX-M}$ genes. Summary of the scatter plots in Supplementary Figure 7, similar to the bottom of Supplementary Figure 4.
Source data are provided as a Source Data file.

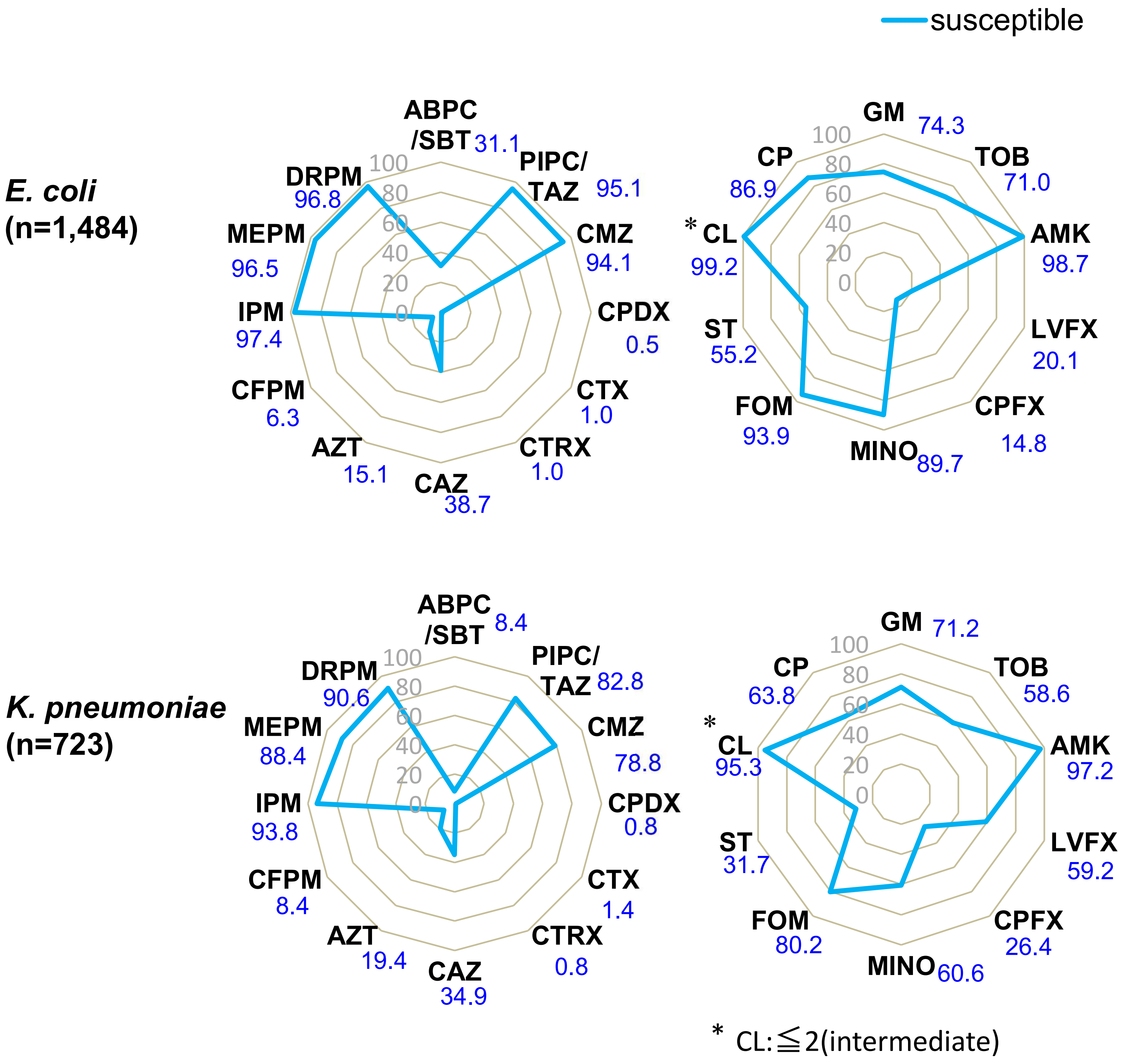

Supplementary Figure 9. Antimicrobial susceptibility profiles of *E. coli* and *K. pneumoniae* strains harboring ESBL genes. The left side displays susceptibility to β-lactam drugs.

Supplementary Figure 10

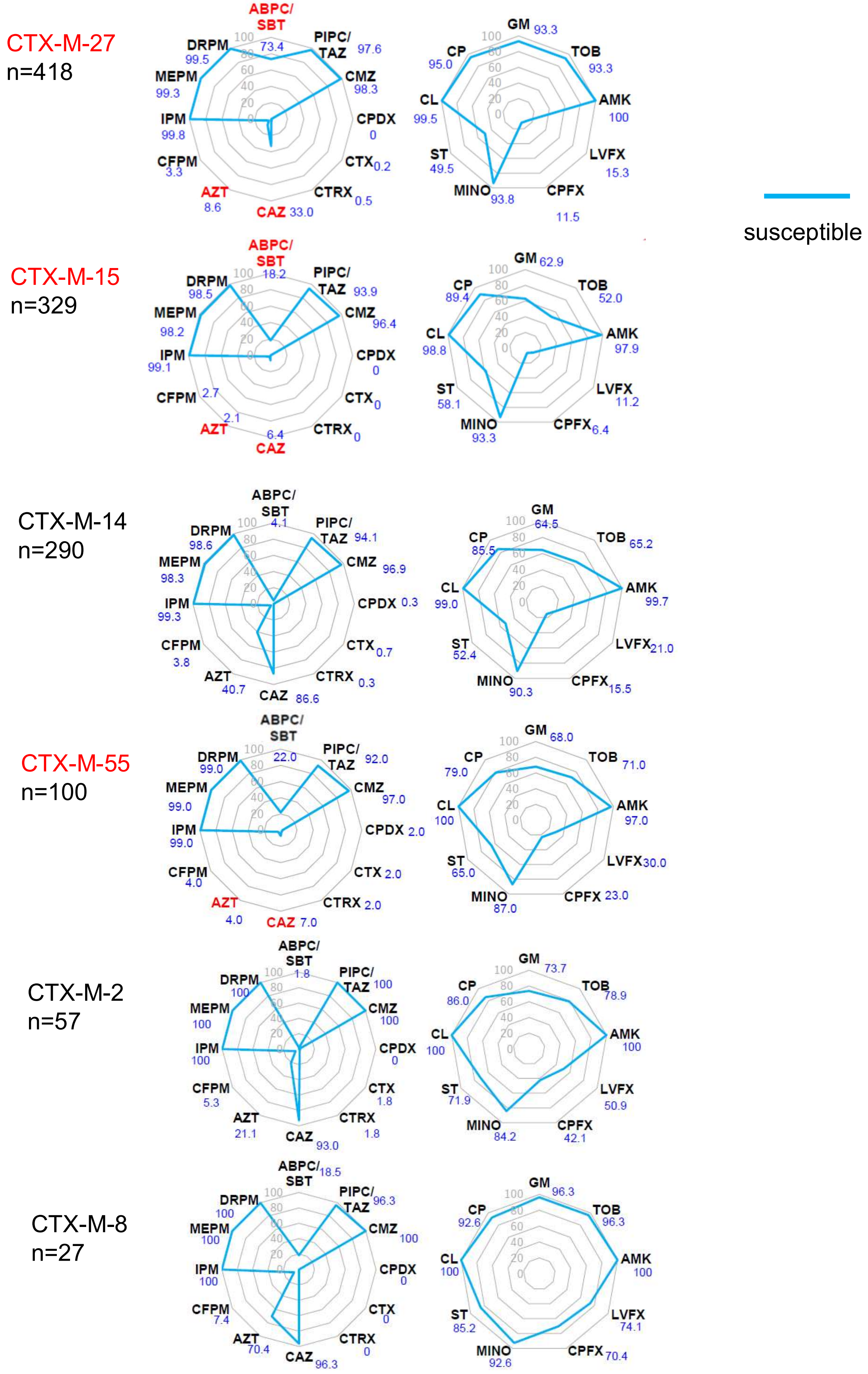

Supplementary Figure 10. Antimicrobial susceptibility profiles of *E. coli* stratified by *bla*$_{\text{CTX-M}}$ genes. Strains harboring carbapenemase genes, AmpC β-lactamase genes, *bla*$_{\text{OXA}}$, and multiple *bla*$_{\text{CTX-M}}$ were excluded.

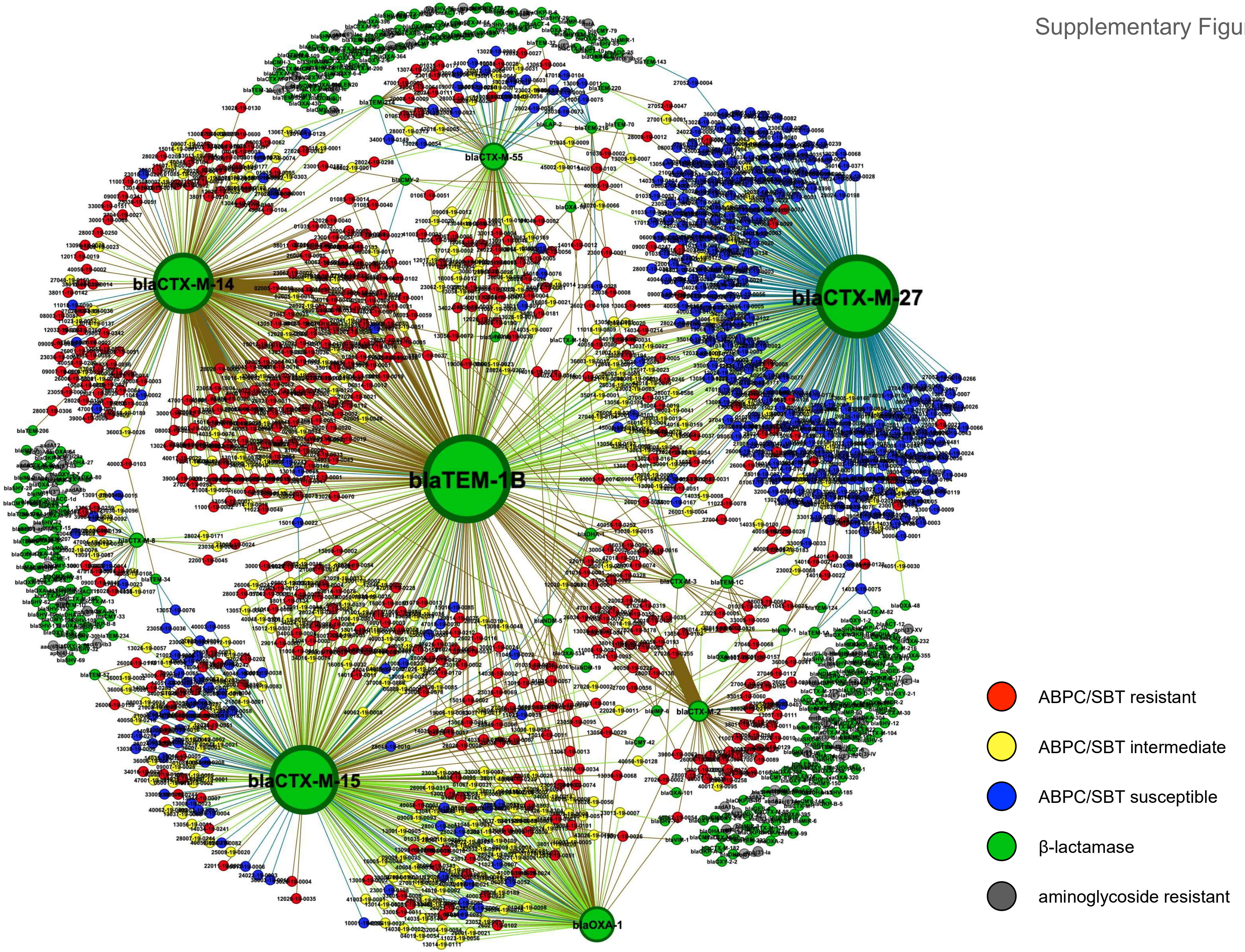

Supplementary Figure 11. Relationship between presence of either $\textit{bla}_{\text{TEM-1B}}$, $\textit{bla}_{\text{CTX-M27}}$, $\textit{bla}_{\text{CTX-M14}}$, $\textit{bla}_{\text{CTX-M15}}$, or $\textit{bla}_{\text{CTX-M55}}$ and resistant/intermediate/susceptible categories to ABPC/SBT. Each dot corresponds to a strain, with different colors indicating different antimicrobial susceptibilities as defined on the right. Source data are provided as a Source Data file.

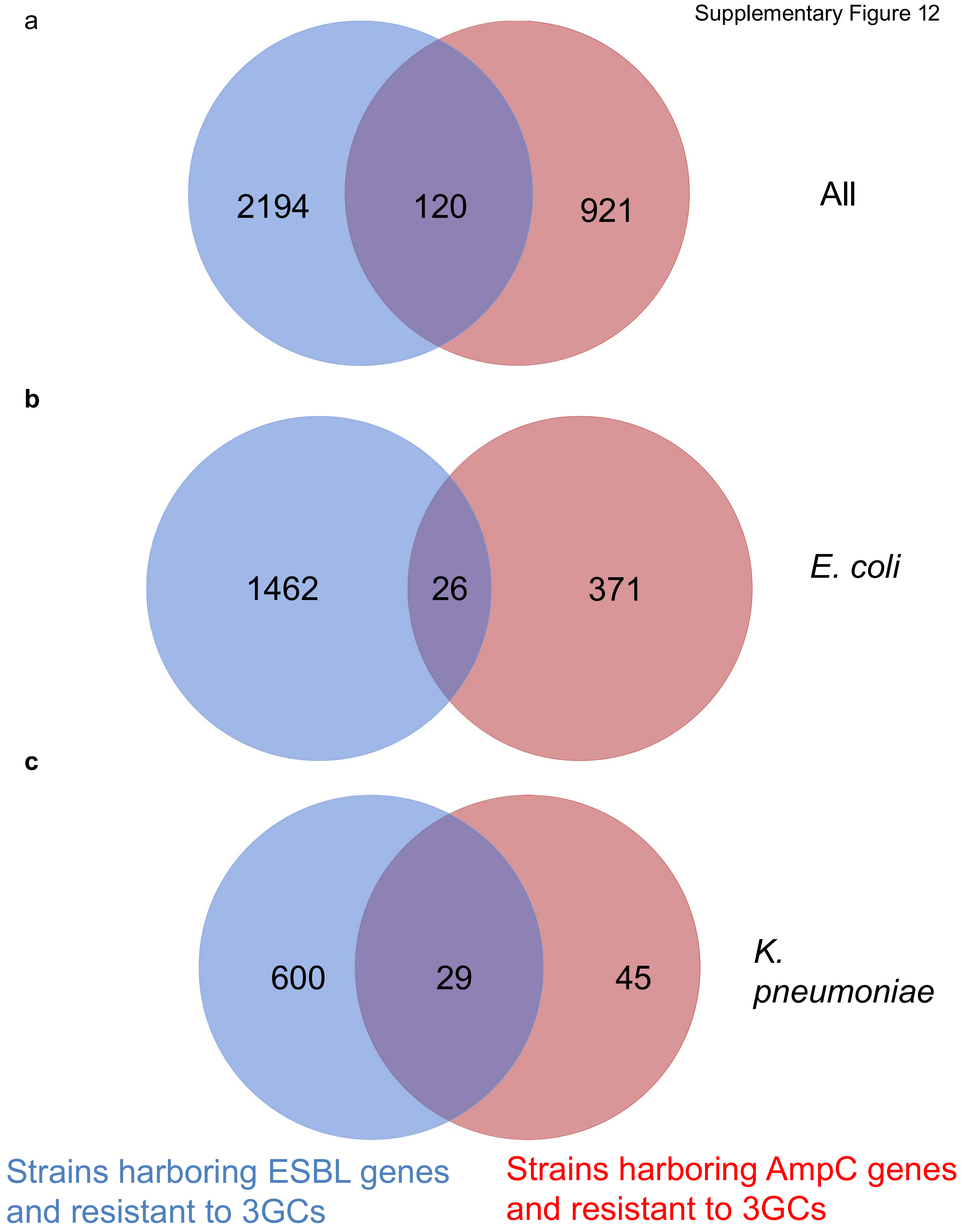

Supplementary Figure 12. Venn diagram showing overlap between 3GC-resistant strains harboring ESBL genes and those harboring AmpC beta-lactamase genes among (a) all, (b) *E. coli*, and (c) *K. pneumoniae* strains. The number of strains is indicated for each section in the diagram.

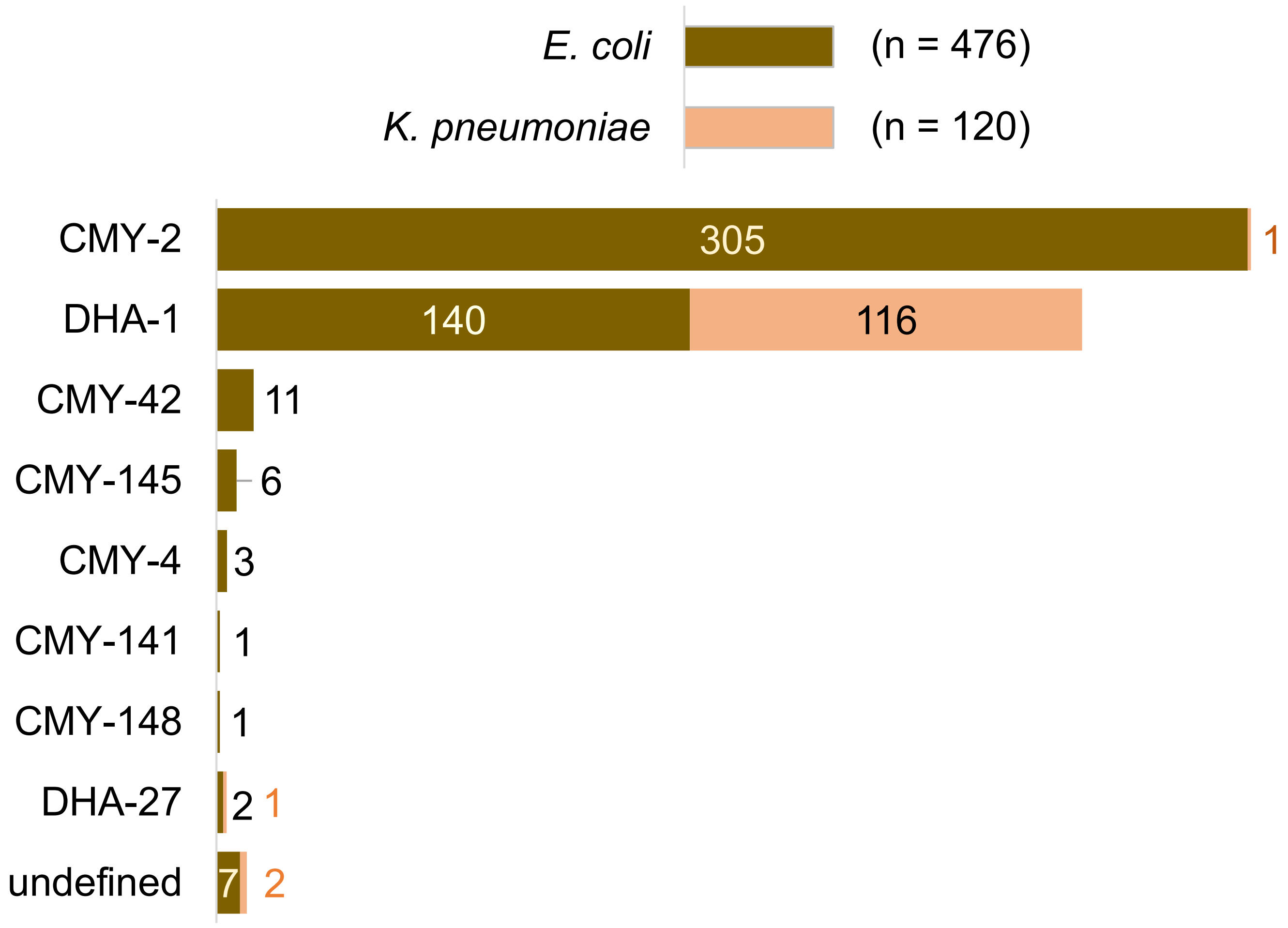

Supplementary Figure 13. Breakdown of AmpC beta-lactamase genes detected in *E. coli* and *K. pneumoniae*, respectively.
The number of strains carrying the gene is indicated for each bar.

E. coli

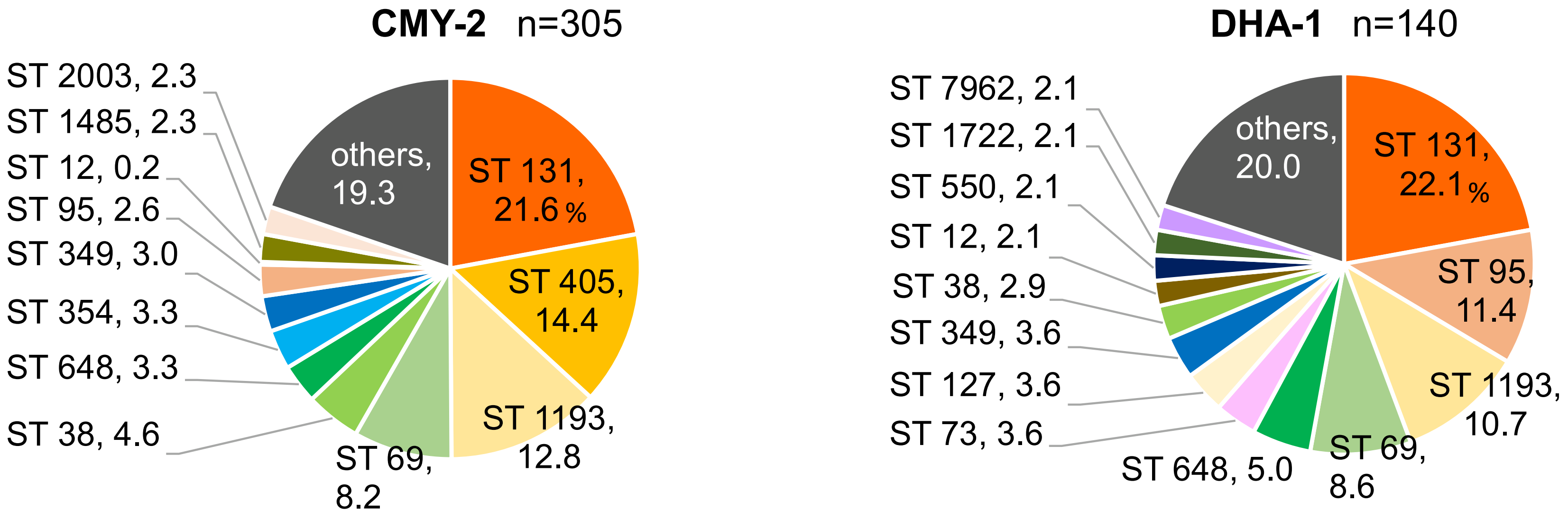

CMY-42 n=11 ST 131:4, ST 354:1, ST 410: 1, ST 448:1, ST 624:1, ST 648:1, ST 1193:1, ST 2659:1

CMY-145 n=6 ST 167:5, ST 2659:1

CMY-4 n=3 ST 131:1, ST 405:1, ST 648:1

CMY-141 n=1 ST 648:1

CMY-148 n=1 ST 648:1

DHA-27 n=2
ST 131: 2

Supplementary Figure 14. Breakdown of STs stratified by the major AmpC beta-lactamase genes in *E. coli*. The numbers in the pie chart indicate percentages.

K. pneumoniae

DHA-1 n=116

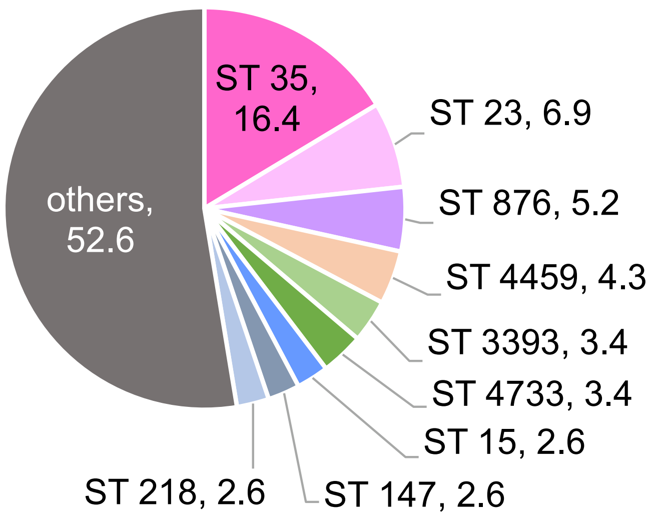

CMY-2 n=1
ST 37 : 1

DHA-27 n=1
ST 14 : 1

Supplementary Figure 15. Breakdown of STs stratified by the major AmpC beta-lactamase genes in *K. pneumoniae*. The numbers in the pie chart indicate percentages.

Supplementary Figure 16

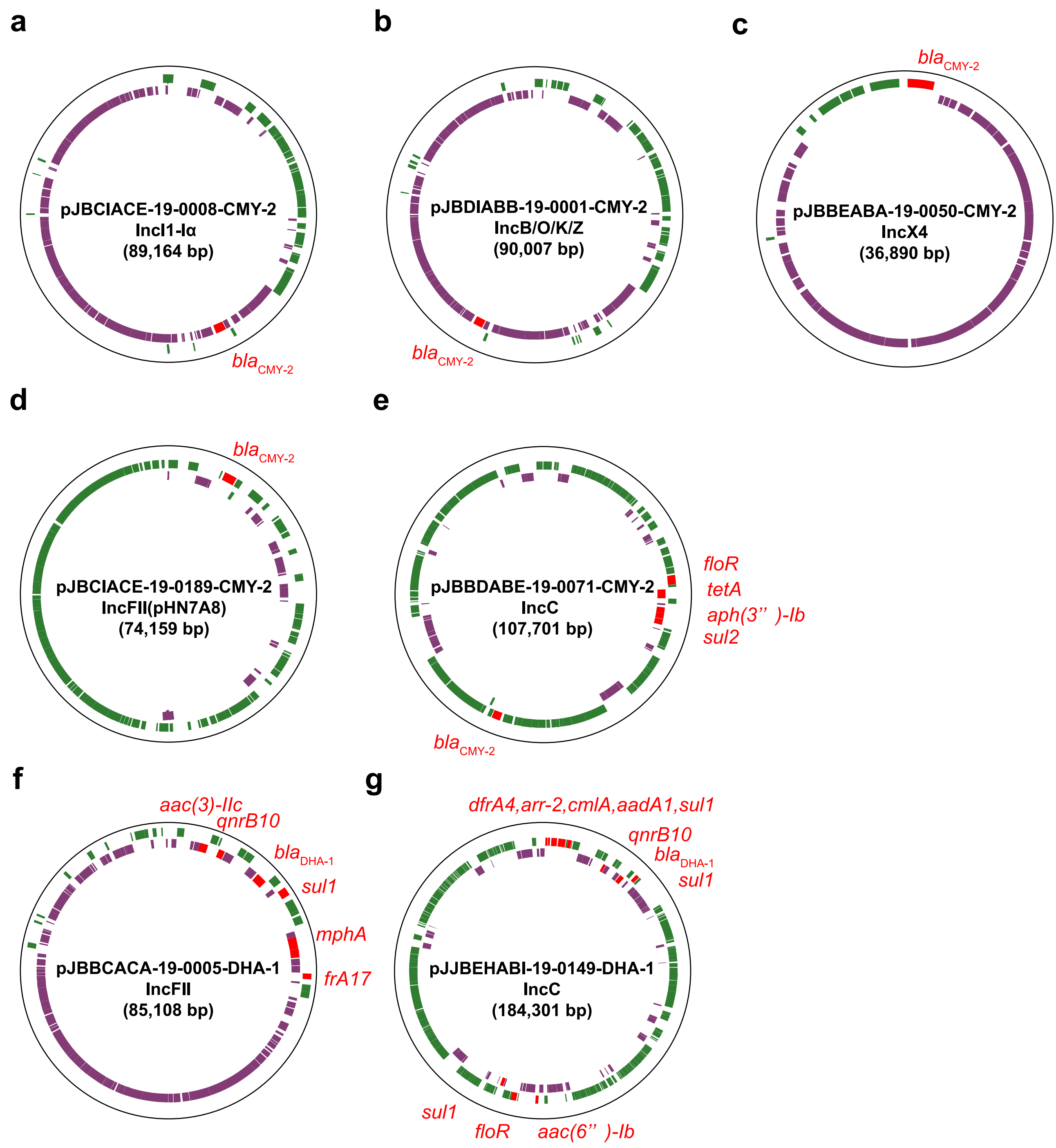

Supplementary Figure 16. Schematic representation of five types of plasmids harboring *bla*$_{\text{CMY-2}}$ and two plasmids harboring *bla*$_{\text{DHA-1}}$. (a) IncI1-Iα plasmid harboring *bla*$_{\text{CMY-2}}$. (b) IncB/O/K/Z plasmid harboring *bla*$_{\text{CMY-2}}$. (c) IncX4 plasmid harboring *bla*$_{\text{CMY-2}}$. (d) IncFII plasmid harboring bla$_{\text{CMY-2}}$. (e) IncC plasmid harboring *bla*$_{\text{CMY-2}}$. (f) IncFII plasmid harboring *bla*$_{\text{DHA-1}}$. (g) IncC plasmid harboring *bla*$_{\text{DHA-1}}$.

Coding regions for antimicrobial resistance gene including *bla*$_{\text{CMY-2}}$ and *bla*$_{\text{DHA-1}}$ are coloured red. Other coding regions are coloured green (clockwise) or purple (anti-clockwise).

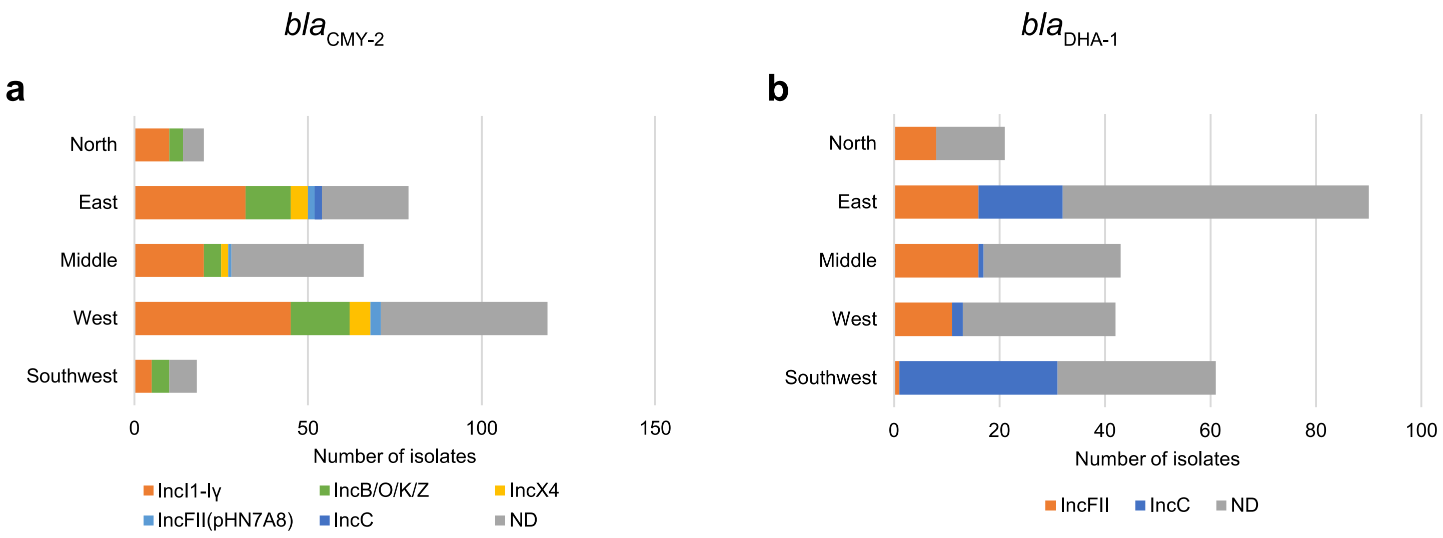

Supplementary Figure 17. Geographical differences in the proportion of strains well aligned by the reference complete plasmid sequences encoding (a) *bla*$_{CMY-2}$ and (b) *bla*$_{DHA-1}$ genes.

Summary of the scatter plots in Supplementary Figure 6, similar to the bottom of Supplementary Figure 4, stratified by the five geographical regions. Source data are provided as a Source Data file.

a

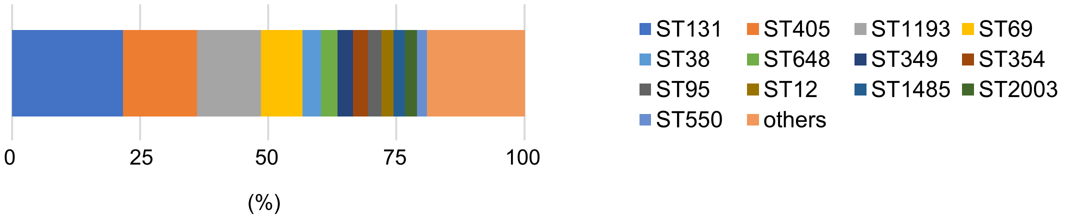

b

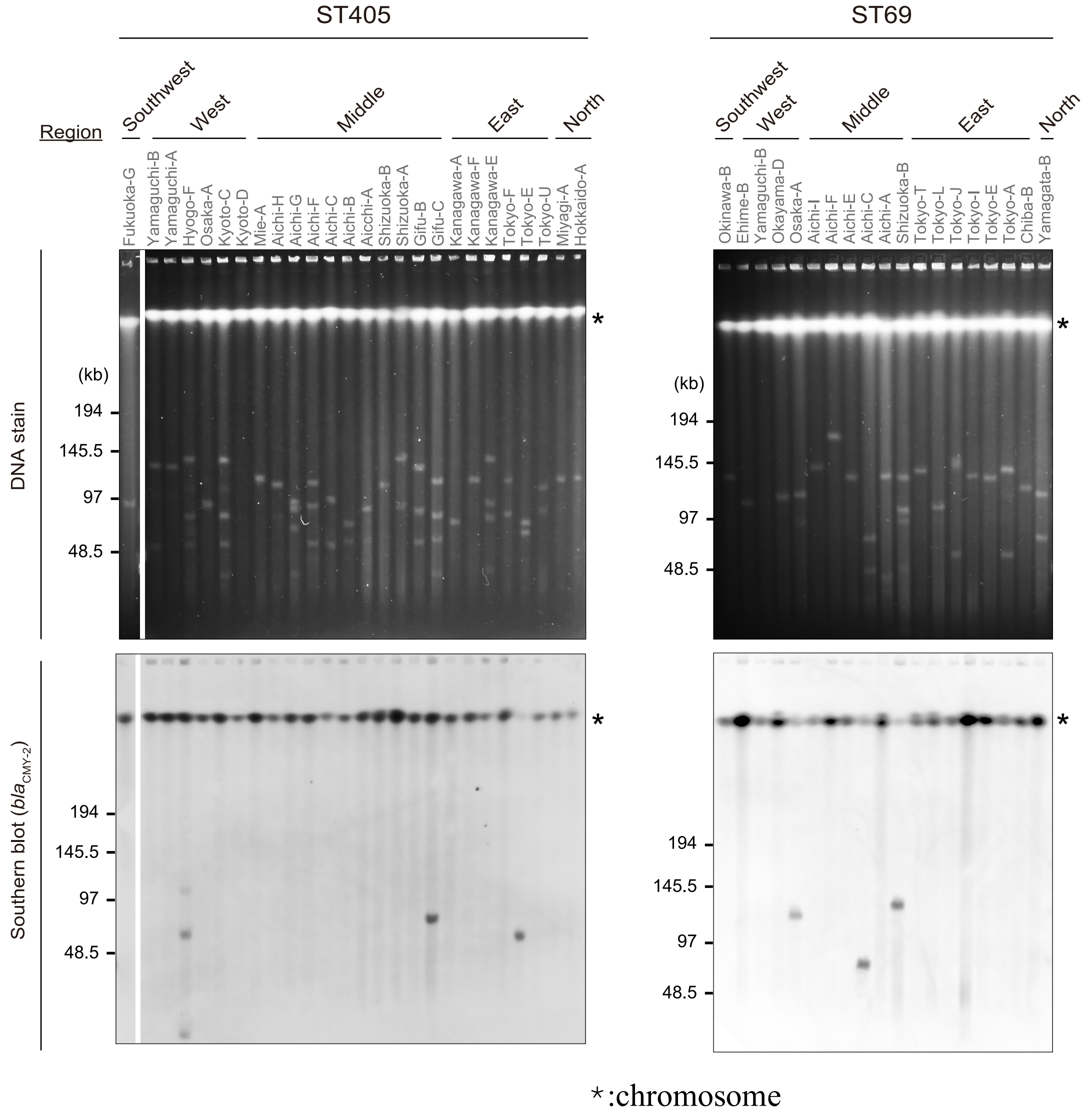

Supplementary Figure 18. (a) Breakdown of STs among *E. coli* strains harboring *bla*$_{\text{CMY-2}}$ genes, and (b) southern blot showing chromosomal *bla*$_{\text{CMY-2}}$ genes in ST405 and ST69.

The star in (b) indicates the chromosomal *bla*$_{\text{CMY-2}}$ gene. Source data are provided as a Source Data file.

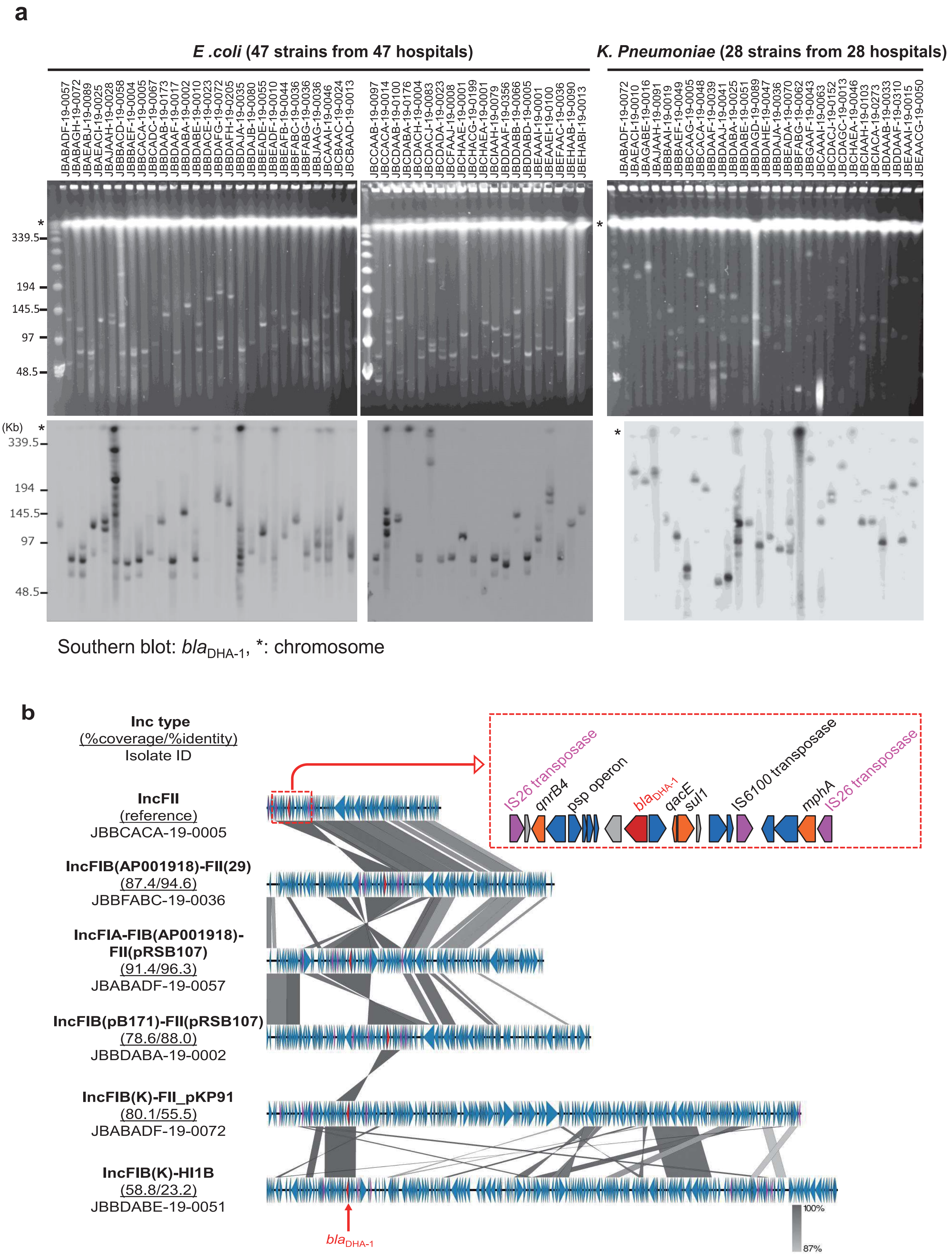

Supplementary Figure 19. Plasmids encoding *bla*$_{DHA-1}$ gene. (a) Southern blot showing diversity of the plasmids. (b) A mobile element encoding *bla*$_{DHA-1}$ and sandwiched between IS26 transposases. The red in (b) indicates blaDHA-1.

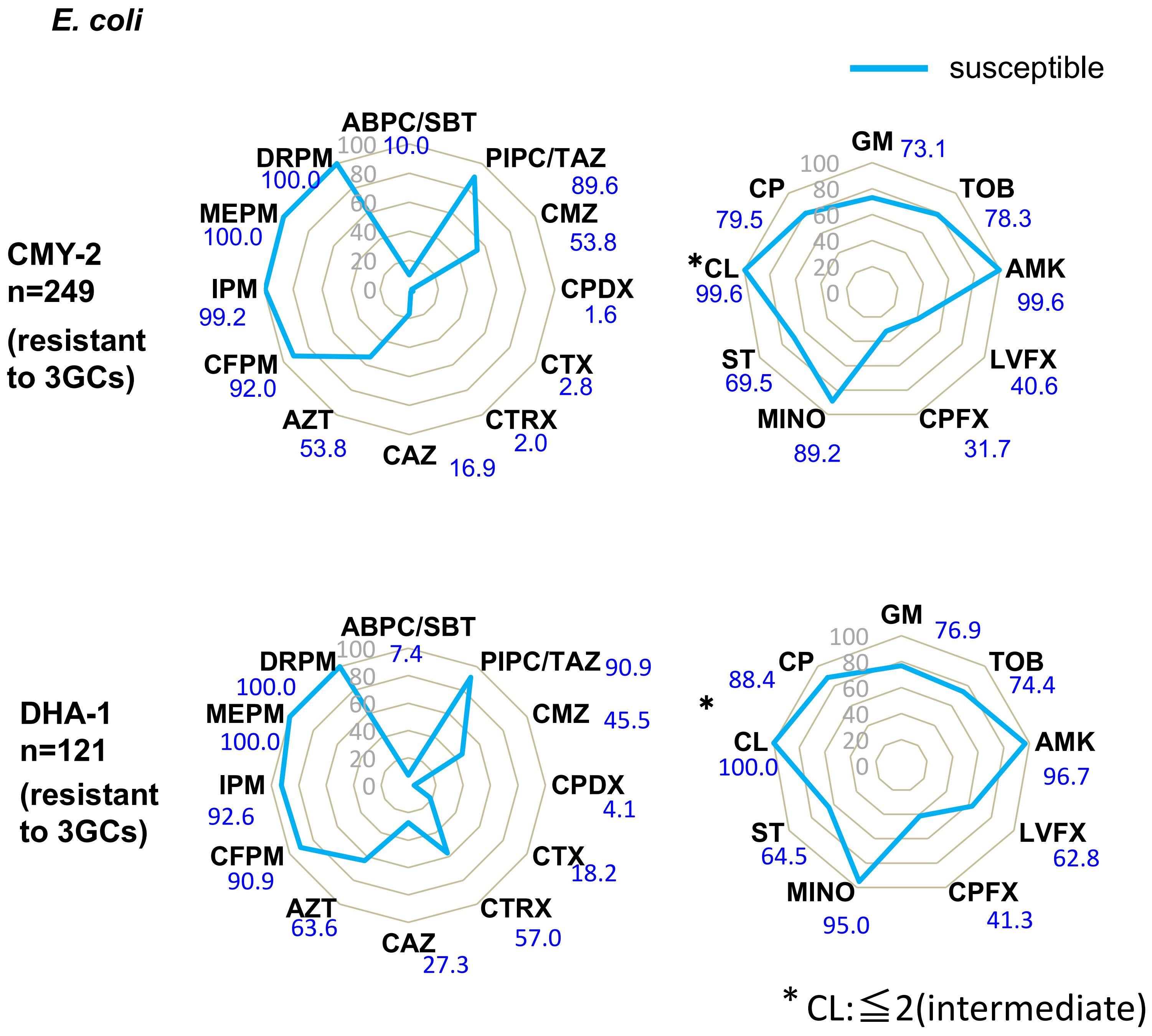

Supplementary Figure 20. Antimicrobial susceptibility profile of *E. coli* strains resistant to 3GCs and harboring AmpC beta-lactamase genes. The left side displays susceptibility to β-lactam drugs.

K. pneumoniae

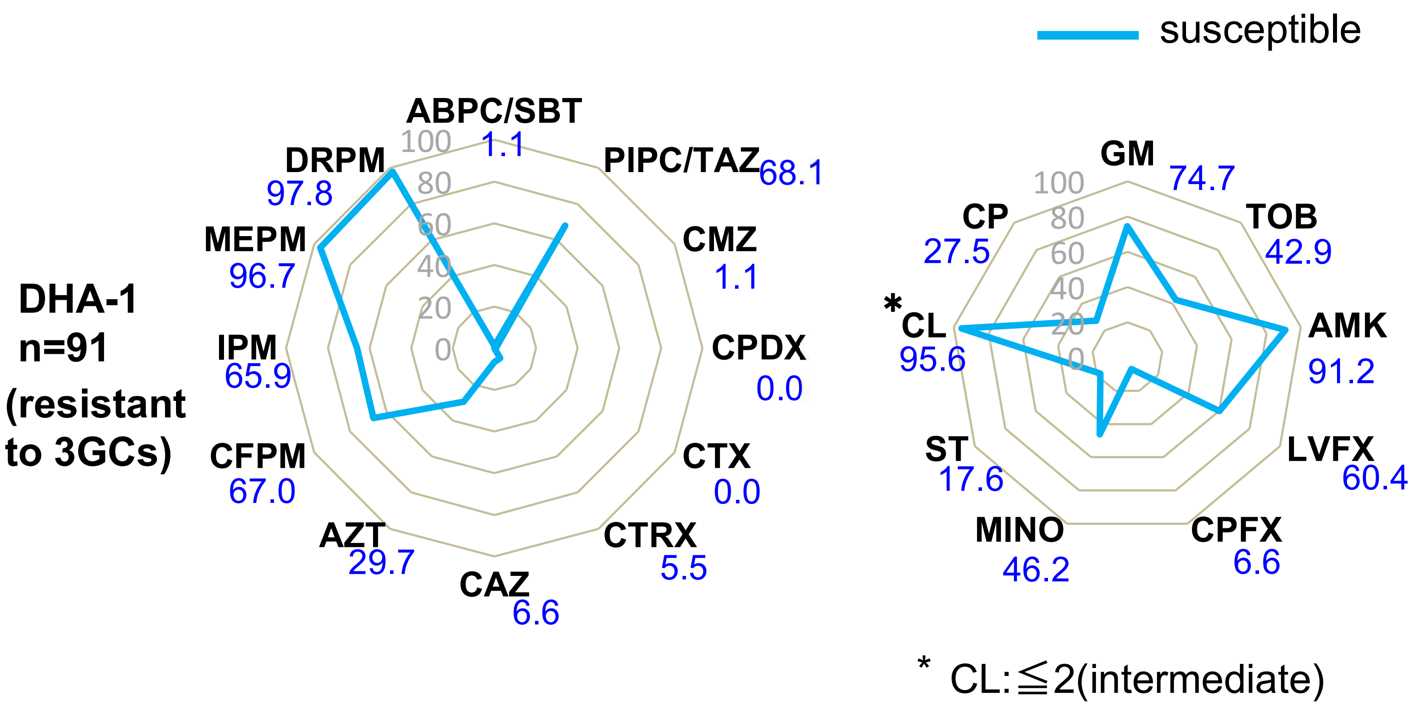

Supplementary Figure 21. Antimicrobial susceptibility profile of *K. pneumoniae* strains resistant to 3GCs and harboring AmpC beta-lactamase genes. The left side displays susceptibility to β-lactam drugs.

Ambler molecular class	B																	A							D			
genome_species	IMP-1	IMP-1+ NDM-1	IMP-6	IMP-11	IMP-19	IMP-60	IMP-66	IMP-97	NDM-1	NDM-4+ OXA181	NDM-5	NDM-5+ OXA-181	NDM-5+ OXA-232	NDM-9	NDM-16b	NDM-19	VIM	GIM-3	KPC-2	GES-4	GES-5	GES-24	IMI-1	IMI-9	FRI-9	OXA-48	OXA-181	OXA-232	Total
Escherichia coli	14	1	8				1				22	1		2	1	1	1		1							1	2	1	57
Klebsiella pneumoniae subsp. *pneumoniae*	39		20		8				1	1			1						2	2		4							78
Klebsiella pneumoniae subsp. *ozaenae*	11		1		3						1									1									17
Klebsiella quasipneumoniae subsp. *quasipneumoniae*	1																												1
Klebsiella quasipneumoniae subsp. *similipneumoniae*	1																					1							2
Klebsiella variicola	6																												6
Klebsiella aerogenes	2										2																		4
Klebsiella grimontii	1																												1
Klebsiella michiganensis	12		2	4																									18
Klebsiella oxytoca	3							1																					4
Citrobacter amalonaticus	1																												1
Citrobacter braakii	1																												1
Citrobacter freundii	20		1																		1	1							23
Citrobacter koseri																						3							3
Citrobacter portucalensis	2																												2
Enterobacter asburiae	10		1			3																1			1				16
Enterobacter bugandensis																							1	2					3
Enterobacter chengduensis	4				1																								5
Enterobacter cloacae	1																						1						2
Enterobacter hormaechei subsp. *hoffmannii*	12		2																										14
Enterobacter hormaechei subsp. *hormaechei*	1																												1
Enterobacter hormaechei subsp. *steigerwaltii*	50		5																										55
Enterobacter hormaechei subsp. *xiangfangensis*	3		1						1																				5
Enterobacter kobei	6																												6
Enterobacter ludwigii	3																												3
Raoultella ornithinolytica			1																										1
Metakosakonia massiliensis	1																		1										2
Mixta calida	1																												1
Providencia rettgeri	2																												2
Serratia marcescens	2																	1			1								4
Serratia nematodiphila	1																												1
Total	211	1	42	4	12	3	1	1	2	1	25	1	1	2	1	1	1	1	4	3	2	10	2	2	1	1	2	1	339

Supplementary Figure 22. Breakdown of carbapenemase genes detected in the present study. Each cell indicates the number of strains carrying each carbapenemase gene.

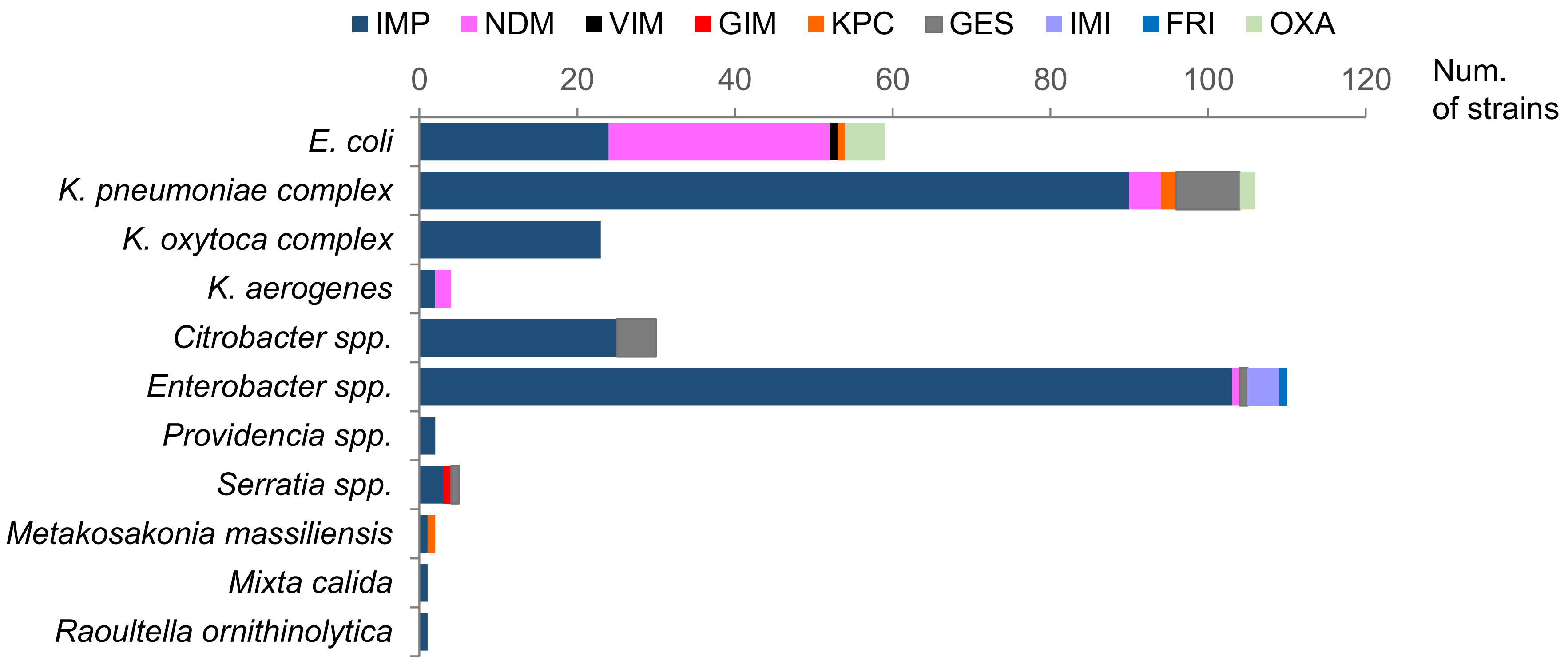

Supplementary Figure 23. Breakdown of carbapenemase genes detected in the present study. The different colors indicate different types of carbapenemase genes.

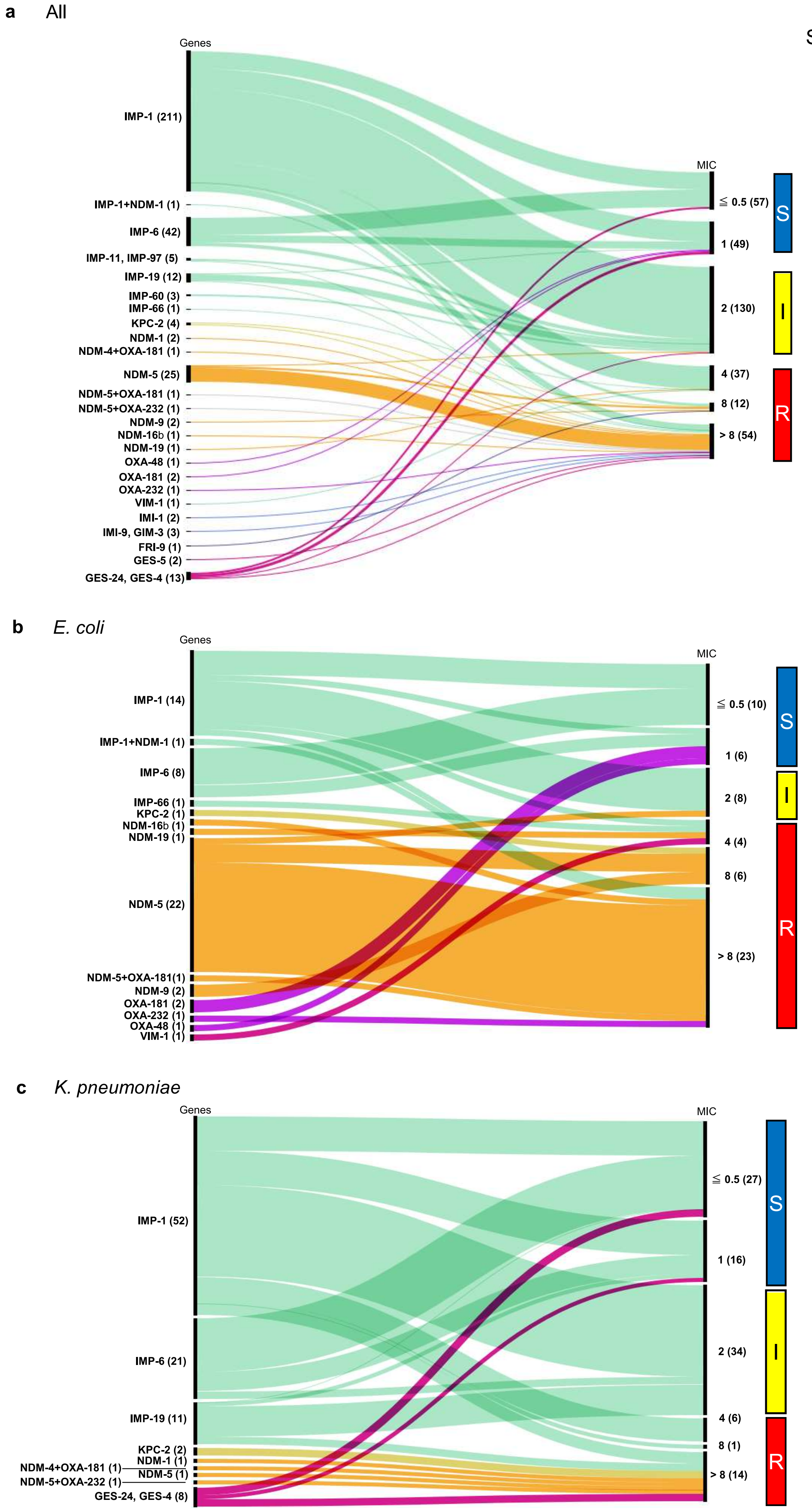

Supplementary Figure 24. Sankey plot summarizing the relationship between carriage of a charbapenemase gene and MICs of imipenem. (a) All. (b) *E. coli*. (c) *K. pneumoniae*
The numbers in the parentheses indicate the number of strains carrying the gene (at the left) and showing the MIC (at the right).

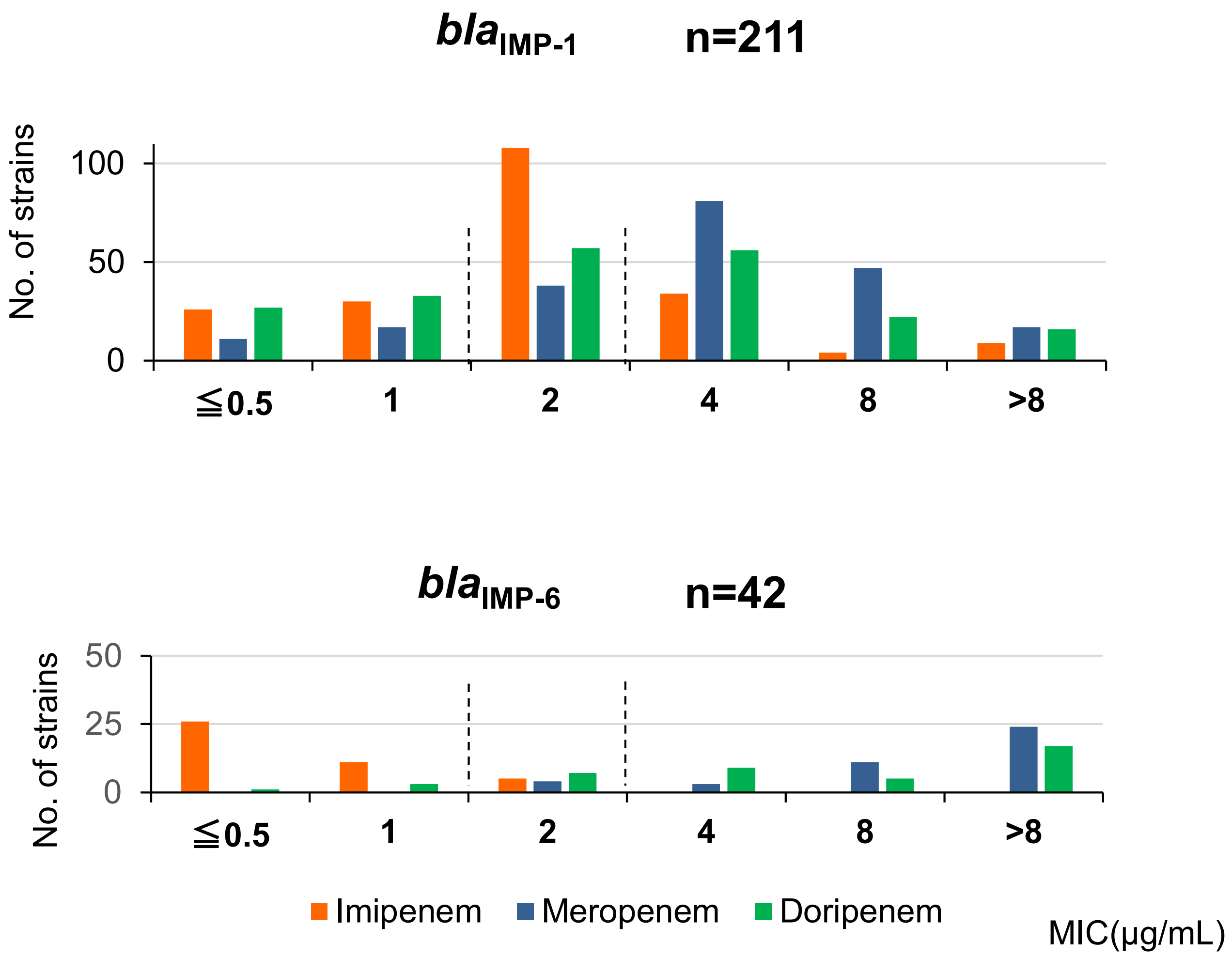

Supplementary Figure 25. Distribution of MIC values among strains harboring $bla_{IMP\text{-}1}$ and $bla_{IMP\text{-}6}$, respectively. The colors orange, blue, and green correspond to imipenem, meropenem, and doripenem, respectively.

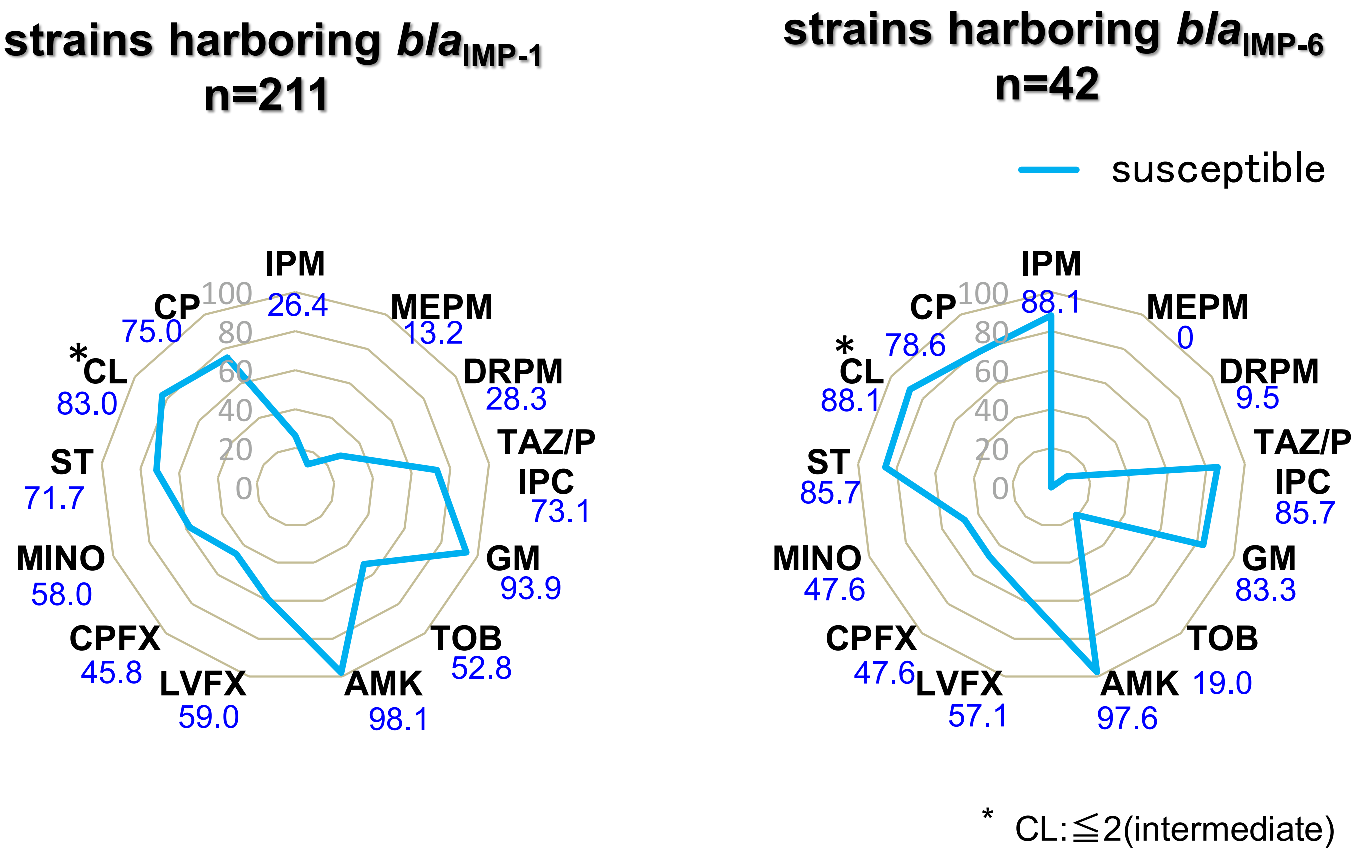

Supplementary Figure 26. Antimicrobial susceptibility profiles of strains harboring *bla*l$_{MP-1}$ and *bla*$_{IMP-6}$, respectively. The left side displays susceptibility to β-lactam drugs.

Supplementary Figure 27

A.

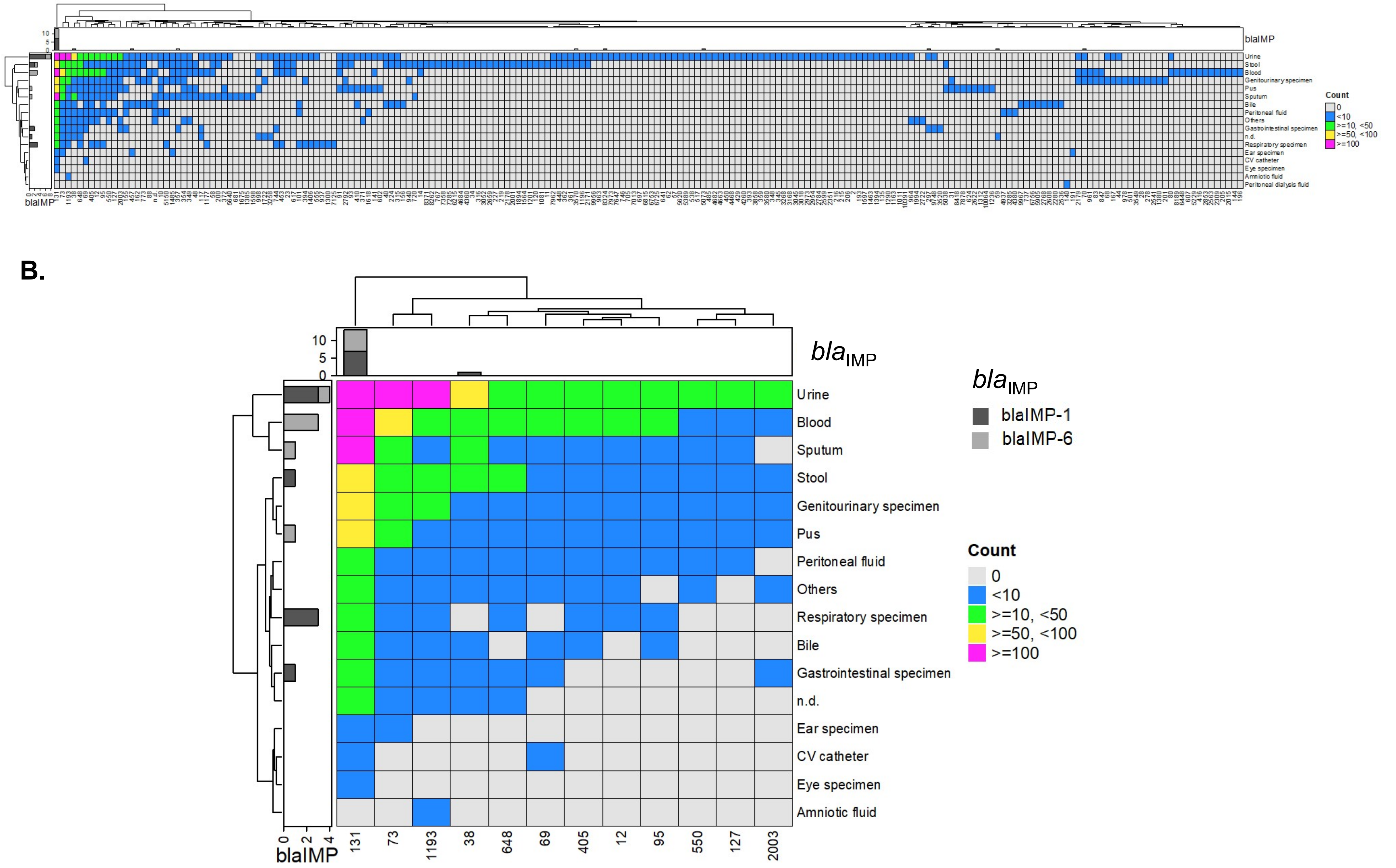

Supplementary Figure 27. Relationship between specimen sources, STs, and the number of strains harboring a *bla*$_{IMP-1}$ or *bla*$_{IMP-6}$ gene.

(A) *E. coli*. (B) *E. coli*, enlargement for STs including > 10 strains.

Source data are provided as a Source Data file.

A.

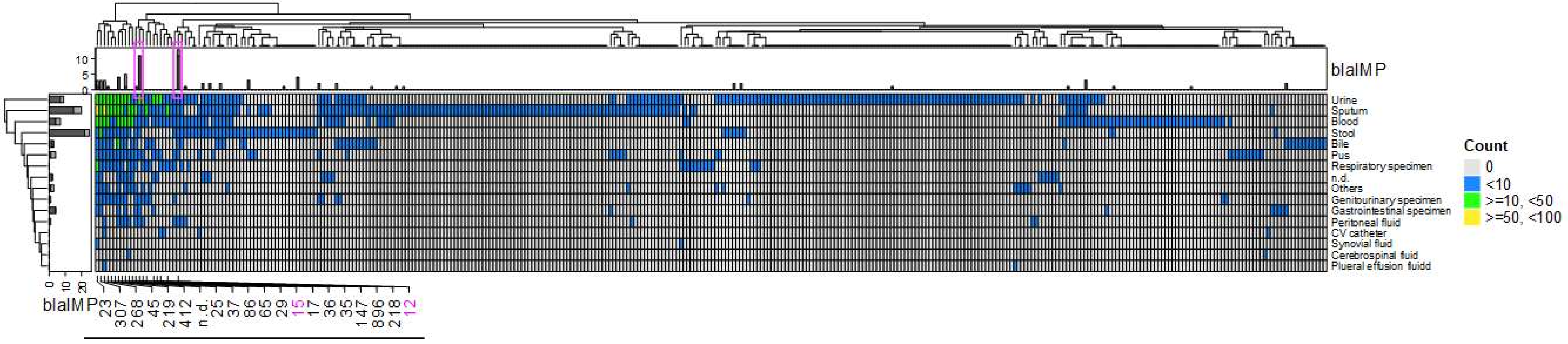
blaIMP
Urine
Sputum
Blood
Stool
Bile
Pus
Respiratory specimen
n.d.
Others
Genitourinary specimen
Gastrointestinal specimen
Peritoneal fluid
CV catheter
Synovial fluid
Cerebrospinal fluid
Plueral effusion fluidd
Count
0
<10
>=10, <50
>=50, <100
blaIMP
23
307
268
45
219
412
n.d.
25
37
86
65
29
15
17
36
35
147
896
218
12

B.

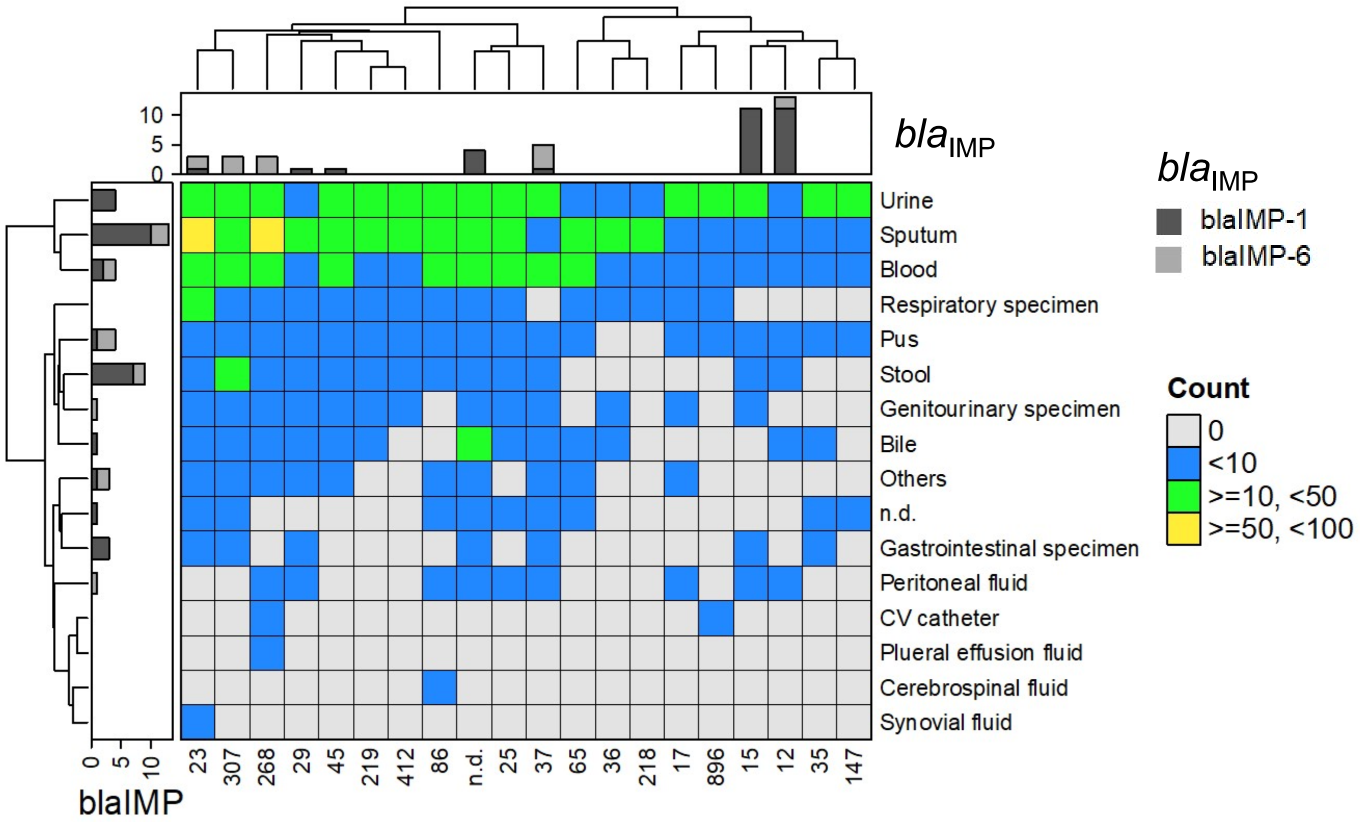
blaIMP
Urine
Sputum
Blood
Respiratory specimen
Pus
Stool
Genitourinary specimen
Bile
Others
n.d.
Gastrointestinal specimen
Peritoneal fluid
CV catheter
Plueral effusion fluid
Cerebrospinal fluid
Synovial fluid
blaIMP
blaIMP-1
blaIMP-6
Count
0
<10
>=10, <50
>=50, <100
blaIMP
23
307
268
29
45
219
412
86
n.d.
25
37
65
36
218
17
896
15
12
35
147

Supplementary Figure 28. Relationship between specimen sources, STs, and the number of strains harboring a *bla*$_{IMP-1}$ or *bla*$_{IMP-6}$ gene.

(A) *K. pneumoniae*. (B) *K. pneumoniae*, enlargement for STs including > 10 strains.

Source data are provided as a Source Data file.

Supplementary Figure 29

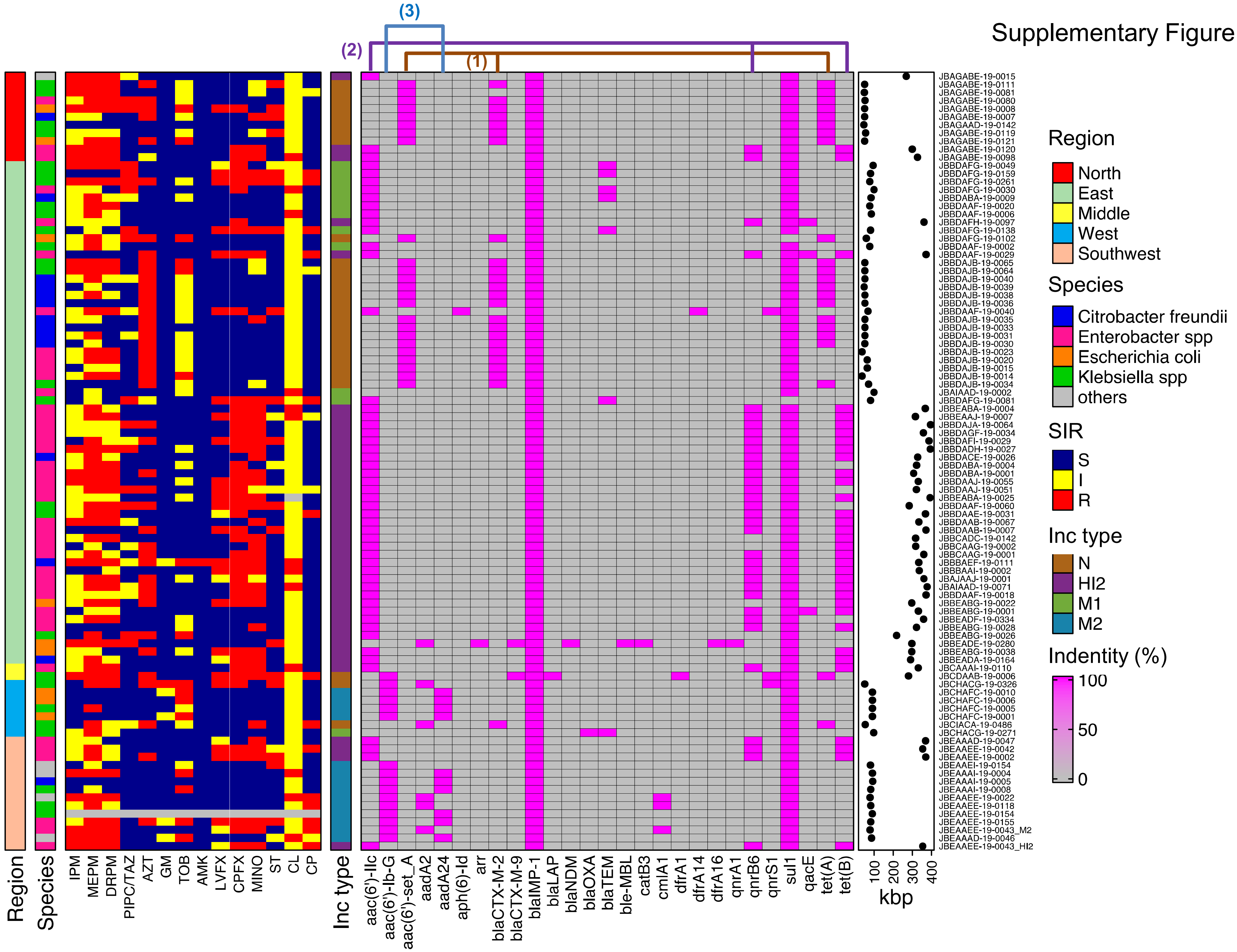

Supplementary Figure 29. Region, species, antimicrobial susceptibility profiles, replicon type, plasmid size, and resistance genes detected across the complete sequences of plasmids harboring *bla*$_{IMP-1}$.

Statistically significant associations between AMR genes are indicated as colored lines at the top and as the numbers corresponding to those in the main text. The colors of the lines at the top correspond to those of Inc type defined at the right: brown is IncN, purple is IncHI2, and light blue is IncM2.Source data are provided as a Source Data file.

Supplementary Figure 30

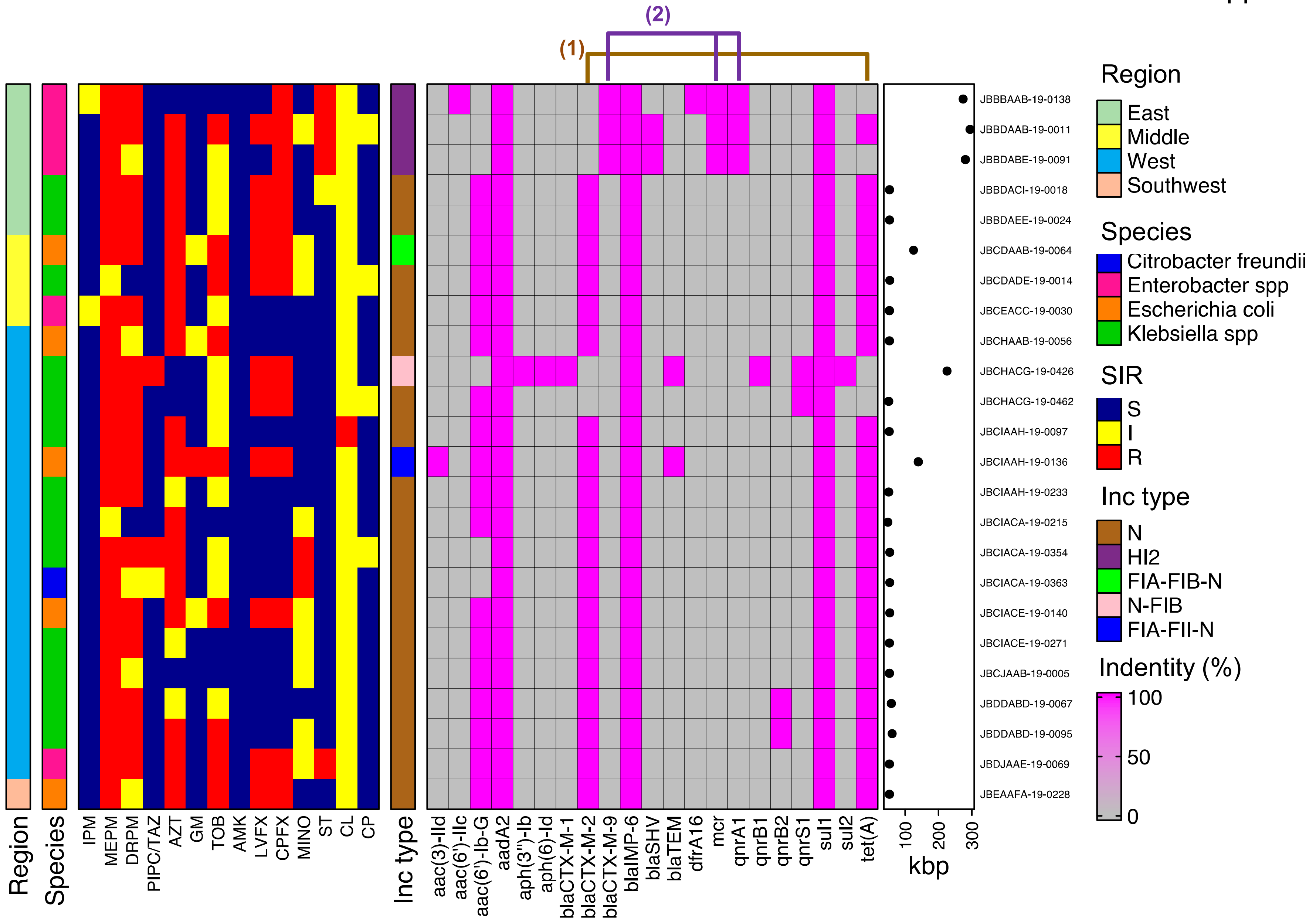

Supplementary Figure 30. Region, species, antimicrobial susceptibility profiles, replicon type, plasmid size, and resistance genes detected across the complete sequences of plasmids harboring *bla*$_{IMP\text{-}6}$.

Statistically significant associations between AMR genes are indicated as colored lines at the top and as the numbers corresponding to those in the main text. The colors of the lines at the top correspond to those of Inc type defined at the right: brown is IncN, and purple is IncHI2.
Source data are provided as a Source Data file.

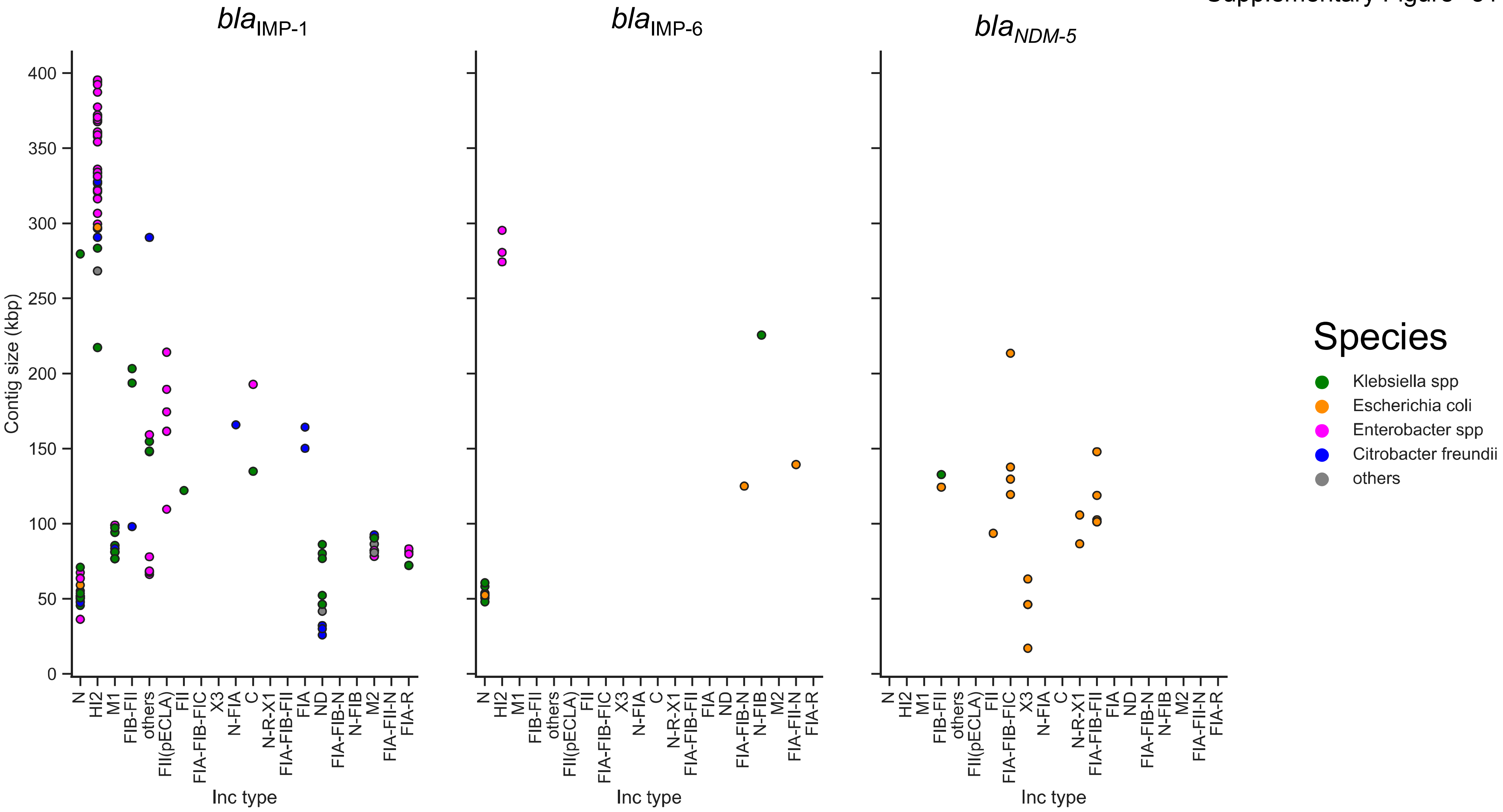

Supplementary Figure 31. Relationship between replicon type and plasmid size for the complete sequences of plasmids harboring *bla*$_{\text{IMP-1}}$, *bla*$_{\text{IMP-6}}$, and *bla*$_{\text{NDM-5}}$, respectively.

The x-axis indicates Inc type; the y-axis indicates plasmid size. The different colors correspond to different species defined at the right. Source data are provided as a Source Data file.

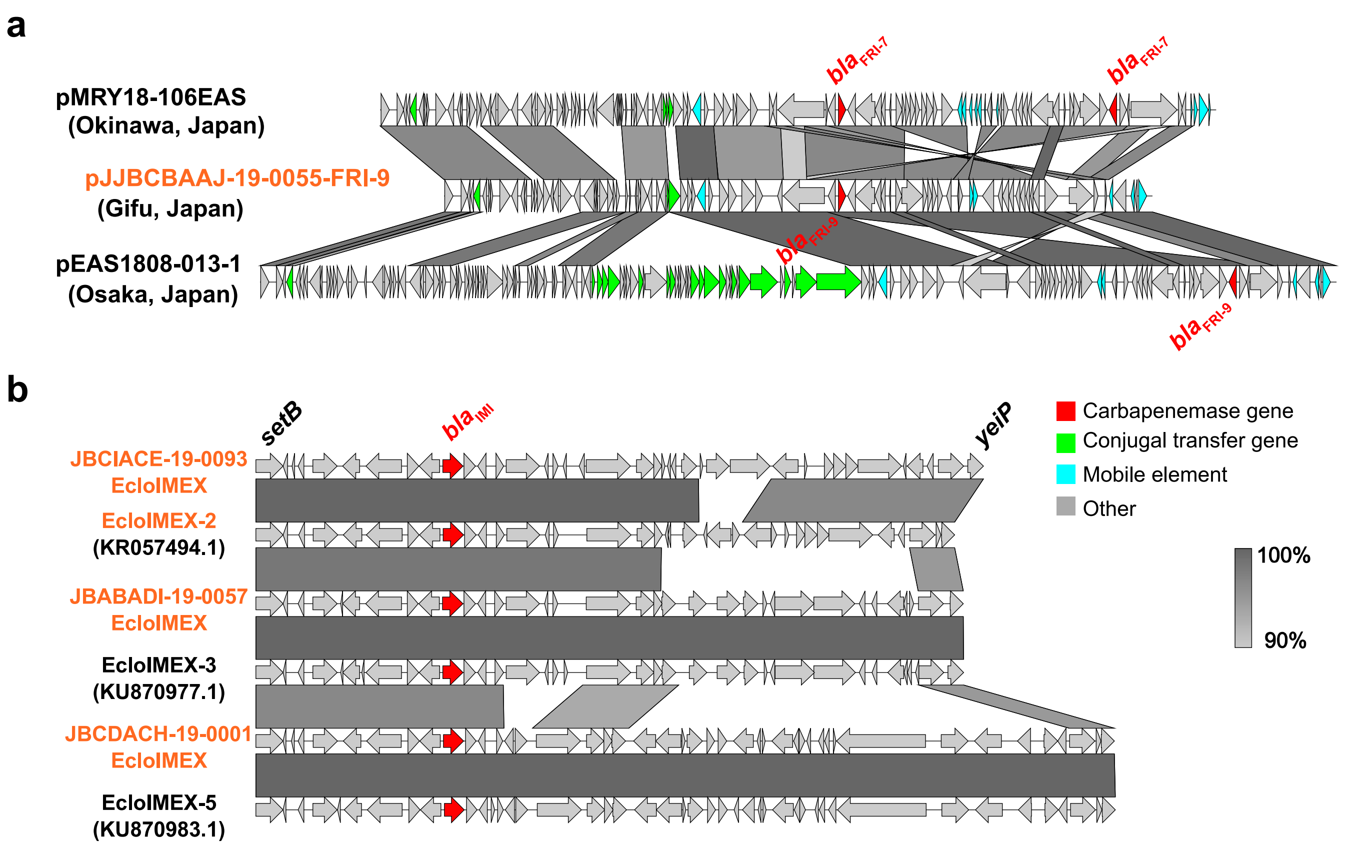

Supplementary Figure 32. Genomic context of the rare carbapenemase genes (a) *bla*$_{\text{FRI-9}}$ and (b) *bla*$_{\text{IMI}}$.

The names of the plasmids in (a) and chromosomal mobile genetic elements harbouring *bla*$_{\text{IMI}}$ (EcloIMEX) in (b) identified in the present study are colored in orange. The carbapenemase genes are colored in red.
