# Supplementary material for: National genomic surveillance integrating standardized quantitative susceptibility testing clarifies antimicrobial resistance in Enterobacterales
Source: Nat Commun. 2023 Dec 5;14:8046. doi: 10.1038/s41467-023-43516-4 (PMC10698200; doi:10.1038/s41467-023-43516-4)
Supplement: Supplementary file 3 — Description of Additional Supplementary Files [file 41467_2023_43516_MOESM3_ESM.pdf]

Description of Additional Supplementary Files

Supplementary Data 1: Classification of *bla*$_{GES}$, *bla*$_{OXA}$, *bla*$_{SHV}$, and *bla*$_{TEM}$ alleles according to BLDB, and frequency counts of each allele. *bla*$_{GES}$ and *bla*$_{OXA}$ alleles are classified as carbapenemase and others, while *bla*$_{SHV}$ and *bla*$_{TEM}$ alleles are classified as ESBL and others, according to the BLDB. The frequency counts of each allele in the present study are shown in the 3rd column.

Supplementary Data 2: Presence or absence of *bla*$_{TEM-1}$, *bla*$_{CTX-M-14}$, *bla*$_{CTX-M-15}$, and *bla*$_{CTX-M-27}$ and ABPC/SBT resistant/intermediate/susceptible phenotype in each of the *E. coli* strains. Source data of Supplementary Figure 10 are shown.

Supplementary Data 3: Breakdown of STs among strains of *E. coli*, *K. pneumoniae*, or *Enterobacter* spp. stratified by carbapenemase genes. The three sheets in the Excel file include data of *E. coli*, *K. pneumoniae*, or *Enterobacter* spp., respectively.

Supplementary Data 4: List of complete sequences of plasmids encoding the three major carbapenemase genes (*bla*$_{IMP-1}$, *bla*$_{IMP-6}$, and *bla*$_{NDM-5}$) reconstructed by long-read sequencing in the present study. Nucleotide sequences of these plasmids are available at https://figshare.com/articles/dataset/Complete_plasmid_sequences_encoding_blaIMP-1_blaIMP-6_and_blaNDM-5/22084769

Supplementary Data 5: Range of MIC values for each antimicrobial drug measured in this study. The NegMIC NF1J panel and NegMIC 3.31E panel were used, and the range of MIC values of each panel is shown in columns D and E, respectively.

Supplementary Data 6: Summary of genome sequence data and the metadata for each isolate. The presence or absence of major antimicrobial resistance genes, as well as other beta-lactamase genes for each isolate, is shown in columns AN to BI. MICs, measured again at the National Institute of Infectious Diseases for each antimicrobial drug, are shown in columns F to AK. The abbreviations of the antimicrobial drugs are listed in Supplementary dataset 5.
