# Supplementary material for: National genomic surveillance integrating standardized quantitative susceptibility testing clarifies antimicrobial resistance in Enterobacterales
Source: Nat Commun. 2023 Dec 5;14:8046. doi: 10.1038/s41467-023-43516-4 (PMC10698200; doi:10.1038/s41467-023-43516-4)
Supplement: Supplementary file 10 — Reporting Summary [file 41467_2023_43516_MOESM10_ESM.pdf]

nature portfolio

Corresponding author(s): Motoyuki Sugai

Last updated by author(s): Sep 12, 2023

Reporting Summary

Nature Portfolio wishes to improve the reproducibility of the work that we publish. This form provides structure for consistency and transparency in reporting. For further information on Nature Portfolio policies, see our Editorial Policies and the Editorial Policy Checklist.

Statistics

For all statistical analyses, confirm that the following items are present in the figure legend, table legend, main text, or Methods section.

n/a	Confirmed	
☐	☒	The exact sample size (*n*) for each experimental group/condition, given as a discrete number and unit of measurement
☐	☒	A statement on whether measurements were taken from distinct samples or whether the same sample was measured repeatedly
☐	☒	The statistical test(s) used AND whether they are one- or two-sided<br>*Only common tests should be described solely by name; describe more complex techniques in the Methods section.*
☒	☐	A description of all covariates tested
☒	☐	A description of any assumptions or corrections, such as tests of normality and adjustment for multiple comparisons
☒	☐	A full description of the statistical parameters including central tendency (e.g. means) or other basic estimates (e.g. regression coefficient) AND variation (e.g. standard deviation) or associated estimates of uncertainty (e.g. confidence intervals)
☐	☒	For null hypothesis testing, the test statistic (e.g. *F*, *t*, *r*) with confidence intervals, effect sizes, degrees of freedom and *P* value noted<br>*Give P values as exact values whenever suitable.*
☒	☐	For Bayesian analysis, information on the choice of priors and Markov chain Monte Carlo settings
☒	☐	For hierarchical and complex designs, identification of the appropriate level for tests and full reporting of outcomes
☒	☐	Estimates of effect sizes (e.g. Cohen's *d*, Pearson's *r*), indicating how they were calculated

Our web collection on statistics for biologists contains articles on many of the points above.

Software and code

Policy information about availability of computer code

Data collection	no software was used
Data analysis	fastp (v0.20.0); DFAST_QC (v0.2.4); shovill (v1.0.4); mlst (v2.16.1); blastn (v2.12.0+); diamond (v2.0.6); AMRFinderPlus (v3.11.14); abricate (v1.0.1); MOB-typer (v3.0.0); EasyFig; Gephi; Staramr (v0.9.1); ForceAtlas2; FastTree (v2.1.11); R 4.1.2 and DescTools package; Microreact; RawGraph; Custom codes were used as explained at https://github.com/bioprojects/JARBS-GNR/

For manuscripts utilizing custom algorithms or software that are central to the research but not yet described in published literature, software must be made available to editors and reviewers. We strongly encourage code deposition in a community repository (e.g. GitHub). See the Nature Portfolio guidelines for submitting code & software for further information.

Data

Policy information about availability of data

All manuscripts must include a data availability statement. This statement should provide the following information, where applicable:

- Accession codes, unique identifiers, or web links for publicly available datasets
- A description of any restrictions on data availability
- For clinical datasets or third party data, please ensure that the statement adheres to our policy

The metadata for each isolate, including MLST, MIC, and genetic polymorphisms, are summarized in Supplementary Table 65. Phylogenetic trees together with metadata visualized using Microreact are available at https://microreact.org/project/piQLyJmufXmM7gwCYzw6eN-microreactecoli3158up2022-04-25 for E. coli

(Supplementary Figure 3) and https://microreact.org/project/2RRmtHGa74444NZHPivC4h-microreactkp1240up2022-04-26 for K. pneumoniae (Supplementary Figure 4). BLDB was used for classification of carbapenemase-type genes and others among blaOXA and blaGES, as well as that of ESBL type genes and others among blaTEM and blaSHV. The 25 complete plasmid sequences encoding blaCTX-M-2, blaCTX-M-14, blaCTX-M-27, blaCTX-M-8, blaCTX-M-3, blaCTX-M-15, or blaCTX-M-55 were deposited in DDBJ under accession numbers DRA014676 to DRA014704 (BioSample accession numbers SAMD00521005 to SAMD00521033, respectively). The raw short- and long-read data of the newly sequenced strains were deposited at DDBJ under the BioProject accession number PRJDB10842 (https://www.ncbi.nlm.nih.gov/bioproject/?term=PRJDB10842). Source data are provided with this paper.

Research involving human participants, their data, or biological material

Policy information about studies with human participants or human data. See also policy information about sex, gender (identity/presentation), and sexual orientation and race, ethnicity and racism.

Reporting on sex and gender

Use the terms sex (biological attribute) and gender (shaped by social and cultural circumstances) carefully in order to avoid confusing both terms. Indicate if findings apply to only one sex or gender; describe whether sex and gender were considered in study design; whether sex and/or gender was determined based on self-reporting or assigned and methods used.
Provide in the source data disaggregated sex and gender data, where this information has been collected, and if consent has been obtained for sharing of individual-level data; provide overall numbers in this Reporting Summary. Please state if this information has not been collected.
Report sex- and gender-based analyses where performed, justify reasons for lack of sex- and gender-based analysis.

Reporting on race, ethnicity, or other socially relevant groupings

Please specify the socially constructed or socially relevant categorization variable(s) used in your manuscript and explain why they were used. Please note that such variables should not be used as proxies for other socially constructed/relevant variables (for example, race or ethnicity should not be used as a proxy for socioeconomic status).
Provide clear definitions of the relevant terms used, how they were provided (by the participants/respondents, the researchers, or third parties), and the method(s) used to classify people into the different categories (e.g. self-report, census or administrative data, social media data, etc.)
Please provide details about how you controlled for confounding variables in your analyses.

Population characteristics

Describe the covariate-relevant population characteristics of the human research participants (e.g. age, genotypic information, past and current diagnosis and treatment categories). If you filled out the behavioural & social sciences study design questions and have nothing to add here, write "See above."

Recruitment

Describe how participants were recruited. Outline any potential self-selection bias or other biases that may be present and how these are likely to impact results.

Ethics oversight

IRB of the National Institute of Infectious Diseases (approval number: 1251) approved collection of bacterial strains from hospitals participating in the surveillance

Note that full information on the approval of the study protocol must also be provided in the manuscript.

Field-specific reporting

Please select the one below that is the best fit for your research. If you are not sure, read the appropriate sections before making your selection.

☒ Life sciences ☐ Behavioural & social sciences ☐ Ecological, evolutionary & environmental sciences

For a reference copy of the document with all sections, see nature.com/documents/nr-reporting-summary-flat.pdf

Life sciences study design

All studies must disclose on these points even when the disclosure is negative.

Sample size

The sample size of all collected strains (N=23,295) was not determined in advance, but includes all Enterobacterales strains resistant to 3GCs and those satisfying the epidemiological cutoff for screening carbapenemase submitted from the 175 hospitals participating in the surveillance.

Data exclusions

5.143 isolated were selected based on the PCR results to include isolates that 1) were carbapenemase gene-positive, 2) were carbapenemase gene-negative with reduced susceptibility to carbapenems according to antimicrobial susceptibility testing results, 3) were representative in terms of ESBL gene carriage patterns; 4) did not grow in CHROMagar ESBL culture, suggesting other mechanisms underlying resistance to 3GCs.

Replication

We have replicated twice the data analyses using the custom codes according to procedures in https://github.com/bioprojects/JARBS-GNR/

Randomization

This is not relevant because there is no group allocation and between-group comparison in this study.

Blinding

This is not relevant because there is no group allocation and between-group comparison in this study.

Reporting for specific materials, systems and methods

We require information from authors about some types of materials, experimental systems and methods used in many studies. Here, indicate whether each material, system or method listed is relevant to your study. If you are not sure if a list item applies to your research, read the appropriate section before selecting a response.

Materials & experimental systems

n/a	Involved in the study
☒	☐ Antibodies
☒	☐ Eukaryotic cell lines
☒	☐ Palaeontology and archaeology
☒	☐ Animals and other organisms
☒	☐ Clinical data
☒	☐ Dual use research of concern
☒	☐ Plants

Methods

n/a	Involved in the study
☒	☐ ChIP-seq
☒	☐ Flow cytometry
☒	☐ MRI-based neuroimaging
